# *In silico* modelling and characterization of eight blast resistance proteins in resistant and susceptible rice cultivars

**DOI:** 10.1186/s43141-020-00076-0

**Published:** 2020-11-25

**Authors:** R. Chandrakanth, L. Sunil, L. Sadashivaiah, N. S. Devaki

**Affiliations:** 1 Department of Molecular Biology, Yuvaraja’s College, University of Mysore, Mysuru, Karnataka 570005 India; 2 Department of Plant Cell Biotechnology, CSIR-Central Food Technological Research Institute, Mysuru, 570020 India

**Keywords:** In silico analysis, Rice blast, NBS-LRR, Resistance proteins, Homology modeling

## Abstract

**Background:**

Nucleotide-binding site-leucine-rich repeat (NBS-LRR) resistance genes are the largest class of plant resistance genes which play an important role in the plant defense response. These genes are better conserved than others and function as a recognition-based immune system in plants through their encoded proteins.

**Results:**

Here, we report the effect of *Magnaporthe oryzae*, the rice blast pathogen inoculation in resistant BR2655 and susceptible HR12 rice cultivars. Transcriptomic profiling was carried out to analyze differential gene expression in these two cultivars. A total of eight NBS-LRR uncharacterized resistance proteins (RP1, RP2, RP3, RP4, RP5, RP6, RP7, and RP8) were selected in these two cultivars for in silico modeling. Modeller 9.22 and SWISS-MODEL servers were used for the homology modeling of eight RPs. ProFunc server was utilized for the prediction of secondary structure and function. The CDvist Web server and Interpro scan server detected the motif and domains in eight RPs. Ramachandran plot of eight RPs confirmed that the modeled structures occupied favorable positions.

**Conclusions:**

From the present study, computational analysis of these eight RPs may afford insights into their role, function, and valuable resource for studying the intricate details of the plant defense mechanism. Furthermore, the identification of resistance proteins is useful for the development of molecular markers linked to resistance genes.

**Supplementary Information:**

The online version contains supplementary material available at 10.1186/s43141-020-00076-0.

## Background

Rice is the most important food crop in the world and a primary source of food for more than half of the world’s population. The causal agent, *Magnaporthe oryzae* B.C. Couch, has been used for several decades as a model organism for understanding the mechanism underlying the host and fungal pathogen interaction [1]. The most important group of genes that have been used by breeders for disease control is the plant resistance (R) genes. Hence, building up a host resistance gene repertoire is of prime concern [2]. Resistance genes (R) are members of a very large multigene family, and these R genes are distributed throughout the 12 rice chromosomes except for chromosome 3 [3]. The rice plants are protected against the pathogens, upon infection, by inducing defense mechanisms via the induction of hypersensitive response (HR), which occurs via gene-for-gene recognition of a pathogen effector and a rice plant-encoded resistance (R) protein [4]. The majority of rice blast resistance genes encode proteins that have a putative central nucleotide-binding site (NBS) and carboxy-terminal leucine-rich repeats (LRR). These NBS-LRR proteins are divided into two major classes: the first class has an N-terminal domain that shares homology with the mammalian Toll-interleukin-1-receptor (TIR) domain while the second class encodes an amino-terminal coiled-coil motif (CC-NBS-LRR) [5].

Rice blast resistance genes are usually constitutively expressed in plants [6]. Major blast R genes like *Pi-ta, Pi-d3, Pi-b, Pi-k1,* and *RGA* were cloned and studied. These blast R genes encode Nod-like receptor (NLR) family proteins that may directly or indirectly interact with fungal effectors to trigger immunity [7].

Reports on structural studies of proteins encoded by blast resistance genes are scanty. Pita and pi54 are the examples of the characterized proteins, and other than these, relatively little is known about the downstream interacting partners of plant NBS-LRR proteins [5, 8].

Crystal structures of mammalian NBS and LRR domains are taken as templates for homology-modeling approaches as complete structures are not available for plant NBS-LRR proteins [9]. NBS-LRR proteins are known to be involved in defense mechanisms in plants. However, other functions carried out by these proteins and their mechanism of action have not been elucidated well. There are many reported protein sequences with functions yet to be experimentally confirmed. These uncharacterized proteins offer a potential for finding numerous applications as biological markers. This can be achieved by using various computational approaches to predict the three-dimensional structure and function of target proteins. Homology modeling is the most accurate method for the structure prediction of uncharacterized protein [10].

In the present study, we modeled eight blast resistance proteins obtained by in silico approach, which are expressed during host-pathogen interaction, predicted their diverse structures, and identified the different domains and binding sites. Structural analysis of these resistance proteins is important for understanding the interaction between *Avr* effector proteins and resistance proteins which are the hallmark of plant defense mechanism.

## Methods

### Plant materials and inoculation

The seeds of resistant BR2655 and susceptible HR12 rice cultivars are collected from Zonal Agricultural Research Station, V.C. Farm, Mandya. These seeds were surfaces sterilized and grown in a greenhouse in a culture chamber (14 h light/10 h dark at temperature 28 ± 1 °C) for 20 days. The rice seedlings were sprayed with conidia at an inoculum concentration of 1 × 10^5^ cells per milliliter containing 0.1% Tween-20. Seedlings which were mock inoculated with 0.1% Tween-20 solution served as control. After inoculation, the leaves were collected separately at 24 h intervals and immediately frozen in liquid nitrogen, and then stored at − 80 °C.

### RNA isolation

Total RNA from rice plant tissues was extracted using the RNeasy Plant Mini Kit (Qiagen, Germany). The quantity and quality of RNA samples were determined using Nanodrop 2000 (Thermo Fisher Scientific) and Agilent 2100 Bioanalyzer (Agilent Technologies). Libraries were prepared using NEBNext® Ultra™ RNA Library Prep Kit for Illumina according to the sample preparation guide. Paired-end sequencing was performed with the TruSeq SBS Kit (Illumina Inc., USA) on Illumina NextSeq 500 (Illumina., USA) (Supplementary Data 2). The sequencing reads were filtered using default parameters for removing the low quality and contaminated reads using readqc analysis. The HQ reads after quality filtering was used for downstream analysis.

### Reference-based assembly and differential gene expression

The overall work carried out in this study is presented in the flow chart (Supplementary Figure 1). High-quality clean reads were mapped to the rice reference genome RGAP7 (http://rice.plantbiology.msu.edu/) using a reference assembly tool of CLC Genomics Workbench and mapping parameters are presented in (Supplementary Table 4).

The rice blast resistance genes expressed in BR2655 and HR12 (upregulated > 3) cultivars were selected based on the keywords “Resistance” and “LRR” (Supplementary Table 2 & 3). Eight genes were shortlisted for the structure determination of their encoded proteins (Supplementary Table 1). Out of these, two genes were chosen from BR2655 and HR12 exclusively. The remaining four genes were selected based on their presence in both the cultivars. The transcripts with log_2_ fold change ≥ 3 (upregulated genes) and ≤ 3 (downregulated genes) with *P* value cutoff of ≤ 0.05 were considered as differentially expressed transcripts at a significant level. Eight proteins expressed by these eight genes were considered for further structural characterization.

### Amino acid sequence retrieval and analysis

Amino acid sequences of NBS-LRR of eight resistance proteins (RP1, RP2, RP3, RP4, RP5, RP6, RP7, and RP8) were retrieved from the Rice Genome Annotation Project. The amino acid sequences of eight resistance proteins were stored as FASTA format sequence and used for further analysis (Supplementary Data 1). The physical and chemical parameters were determined by using the ExPASy Prot Param. The similarity search was performed against the non-redundant database in protein data bank (PDB), and PDB structures were used to search similar structures to that of eight RP proteins using PSI-BLAST tool [11].

### Homology modeling

SWISS-MODEL server (https://swissmodel.expasy.org) [12] and Modeller 9.22 (https://salilab.org/modeller/download_installation.html) [13] programs were used to build and generate the three-dimensional structures of eight resistance proteins. The three-dimensional structures were visualized with the UCSF Chimera program [14].

### Conserved motif structures and phylogenetic analysis of RP proteins

The amino acid sequences of eight resistance proteins were subjected to domain and motif search by using The CDvist Web server [15] and Interpro scan server [16]. The eight resistant protein sequences were aligned for Multiple Sequence Alignment (http://www.ebi.ac.uk/Tools/msa/muscle/) to observe the homology sequence alignment among resistance proteins using ClustalW [17]. The phylogenetic analysis was performed to see the evolutionary relationship among reported resistance genes such as *Pita, Pid3, Pik2, Pib, Pi54, Pik1, RGA4,* and *RGA5* and the eight resistance proteins (RP1 to RP8).

### Structure and function analysis of RP

The secondary structures of eight resistance proteins were predicted by using the RaptorX (http://raptorx.uchicago.edu) prediction server [18] and ProFunc server [19] which use methods such as fold matching, residue conservation, surface cleft analysis, and functional 3D templates [19].

### Validation of RP proteins

The quality of the predicted three-dimensional structure models of eight resistance proteins was analyzed through SAVeS Server (https://services.mbi.ucla.edu/SAVES/) [20] and SuperPose (http://wishart.biology.ualberta.ca/SuperPose/) [21].

## Results

### Differential gene expression analysis

Experiments on disease screening revealed the different stages of resistance between BR2655 and HR12 rice cultivars. BR2655 and HR12 seedlings inoculated with *M. oryzae* (M036) conidial suspension, showed disease scoring 2 (resistant), and 8 (susceptible) respectively based on the IRRI SES scale.

We observed the difference in gene expression profiling in BR2655 and HR12 rice cultivars during infection by *M. oryzae*. In total, we obtained 75.8 and 69.7 million raw reads for BR2655 and HR12 rice cultivars, respectively (Supplementary Data 3 and Supplementary Figure 2). We identified 7577 and 4290 differentially expressed genes (DEG) in the resistant line (R) (BR2655) and susceptible line (S) (HR12), respectively. As per the “LRR” keyword search, 22 transcripts, which are upregulated in BR2655 cultivar were shortlisted and with the “Resistance” keyword search, 36 transcripts were enlisted. Correspondingly, the upregulated transcripts were shortlisted in HR 12 cultivar using the same keyword search, and there were 17 and 38 transcripts, respectively.

### Amino acid sequence retrieval and analysis

The amino acid sequences of eight RPs were retrieved from the RGAP. The eight RPs were analyzed for amino acid composition by the ExPASy ProtParam tool (Table 1). Leucine was the most frequent amino acid present in the sequence in all eight RPs and the percentage of leucine residues ranged from 11.4 to 15.0%. RP5 was found to have the least percentage of leucine content, i.e., 11.5%, and RP2 showed the highest percentage of leucine content, i.e., 15.0%.

**Table 1:** Amino acid composition of eight resistant proteins

Amino acid composition	%							
	RP1	RP2	RP3	RP4	RP5	RP6	RP7	RP8
Alanine (A)	4.9	4.5	5.4	6.2	5.4	5.5	4.9	4.0
Arginine (R)	5.3	4.1	6.1	5.9	5.3	5.5	6.4	7.9
Asparagine (N)	3.4	4.5	4.1	3.8	4.2	4.0	4.5	3.3
Aspartic acid (D)	5.8	5.5	4.5	4.9	7.0	6.0	5.2	6.3
Cysteine (C)	3.3	2.3	2.5	2.7	1.9	2.6	2.4	2.8
Glutamine (Q)	4.1	4.1	4.3	4.0	4.5	4.0	4.3	2.8
Glutamic acid (E)	6.0	7.3	6.7	7.1	10.1	7.8	8.4	6.2
Glycine (G)	5.3	5.9	6.8	4.6	5.1	6.4	5.7	5.7
Histidine (H)	2.6	3.2	2.8	3.0	3.1	2.3	2.4	3.2
Isoleucine (I)	5.3	6.0	5.2	5.4	6.0	5.8	5.9	6.2
**Leucine (L)**	**14.4**	**15.0**	**11.5**	**13.7**	**11.4**	**14.0**	**13.7**	**14.0**
Lysine (K)	5.6	7.5	6.7	5.8	9.7	6.5	6.3	4.5
Methionine (M)	2.6	1.2	1.8	2.6	1.6	1.5	1.7	2.3
Phenylalanine (F)	3.4	4.0	4.0	2.8	2.2	2.8	2.6	3.4
Proline (P)	3.6	3.8	4.1	4.2	3.8	3.9	4.0	3.4
Serine (S)	7.1	7.4	7.4	7.4	5.5	6.8	6.5	9.2
Threonine (T)	5.0	5.0	4.8	4.9	4.2	4.3	4.7	3.8
Tryptophan (W)	2.7	1.2	2.1	2.1	1.0	1.9	1.8	0.8
Tyrosine (Y)	2.8	2.0	3.1	2.2	3.0	1.9	2.1	3.0
Valine (V)	7.1	5.6	6.2	6.7	5.2	6.4	6.3	7.2

Values in bold indicate that all resistant proteins are rich in leucine residues confirming the presence of leucine-rich repeats

PSI-BLAST analyses of eight RPs were performed against non-redundant protein to determine the protein family, and top 4 best BLAST scores were obtained for each RP (Table 2). The sequence identity ranged between 57 and 100% in eight RPs, and the query coverage was in the range of 68–100%.

**Table 2:** Sequence identity and similarity between BCRP and available templates

	Name	Query cover (%)	Identity (%)	Total score	Accession
**RP1**	Hypothetical protein Osl_05235	100	100	1769	EAY77261.1
	NBS-LRR like resistance protein	100	99	1753	ALO70091.1
	Hypothetical protein OsJ_04789	98	99	1749	EEE56018.1
	Putative blight resistance protein RGA1	100	97	1727	BAD87860.1
**RP2**	Predicted: disease RP RGA2-like	99	100	3056	XP_015645850.1
	Hypothetical protein OsI_24856	99	99	3054	EAZ02738.1
	NBS-LRR-like resistance protein	99	99	3030	ALO70120.1
	NBS-LRR-like resistance protein	99	99	3029	ALO70121.1
**RP3**	Os09g0314100 [Oryza sativa japonica Group]	99	95	2926	BAH94489.1
	Hypothetical protein Osj_28823	99	92	2793	EEC84327.1
	Hypothetical protein OsJ_28823	90	99	2757	EEE69434.1
	Os09g0314200 [Oryza sativa Japonica Group]	68	98	2075	BAT07400.1
**RP4**	NB-ARC domain, putative	99	100	2234	AAX95985.1
	Predicted: putative disease RP RGA3	91	100	2044	XP_015615198.1
	Predicted: putative disease RP RGA4	91	84	1670	XP_015697828.1
	Predicted: uncharacterized protein	91	58	1092	XP_014757772.1
**RP5**	Leucine rich repeat family protein, expressed	99	100	2624	ABA94704.2
	Os11g0598500 [Oryza sativa Japonica Group]	96	97	2522	BAF28579.1
	Predicted: uncharacterized protein	96	95	2447	XP_015615610.1
	Hypothetical protein OsJ_34447	71	100	1865	EEE52373.1
**RP6**	Predicted: Putative disease RPRGA4	99	100	2118	XP_015617680.1
	Os11g0763600 [Oryza sativa japonica Group]	94	99	2011	BAH95435.1
	NBS-LRR-like protein	99	93	1901	AAK93796.1
	Predicted: Putative disease RPRGA4	99	90	1826	XP_015617526.1
**RP7**	Predicted: Putative disease RPRGA3	99	100	2122	XP_015616937.1
	Os11g0676050 [Oryza sativa japonica Group]	99	90	3133	BAH95438.1
	Predicted: Putative disease RPRGA4	99	90	1790	XP_015617526.1
	Predicted: Putative disease RPRGA4	99	84	1709	XP_015617680.1
**RP8**	NB-ARC domain containing protein	99	100	1845	ABA96074.2
	Hypothetical protein OsJ_35504	86	96	1503	EAZ19911.1
	Predicted: disease resistance RPP13-like protein 3	95	60	1009	XP_010238635.1
	Predicted: disease resistance RPP13-like protein 3	99	57	998	XP_015698835.1

*RP* resistance protein

### Physico-chemical properties of eight resistance proteins

Physico-chemical parameters like molecular weight, pI, amino acid composition, estimated half-life, and instability index were performed in ExPASy ProtParam. The predicted molecular mass of eight RPs (RP1 to RP8) had 97.8 kDa, 168.3 kDa, 168.2 kDa, 122.7 kDa, 146.2 kDa, 116.4 kDa, 117.4 kDa, and 102.5 kDa, respectively. The isoelectric points (pI) of eight RPs were found to be in the acidic range except for RP3 and RP8, which were slightly basic. The eight RPs showed high aliphatic index values (88.09–103.58), which indicated that the proteins are stable for a broad range of temperatures [22].

### Motifs and phylogenetic analysis

**Fig. 1:**
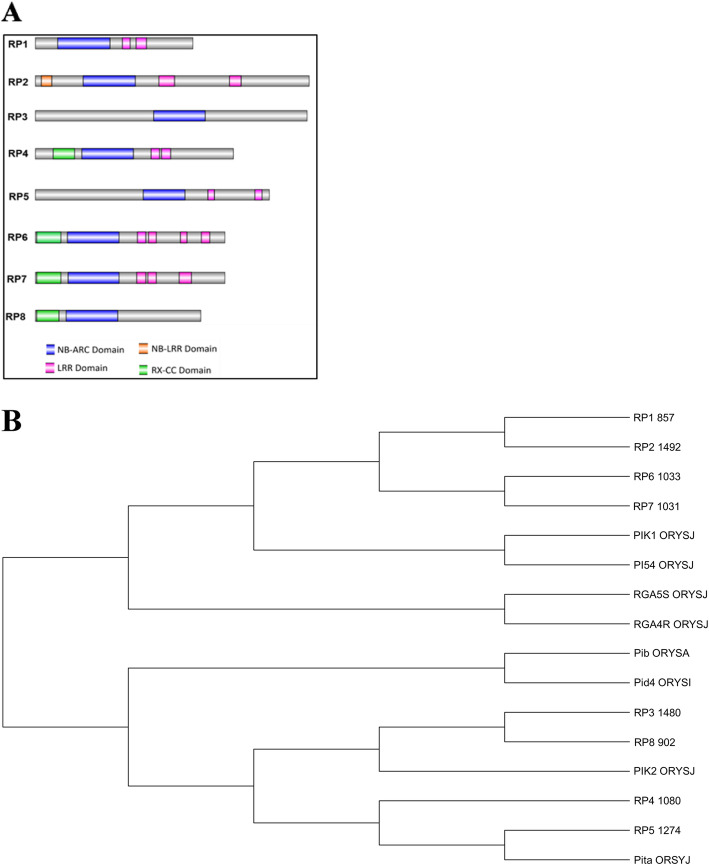
**a** Domain architecture of eight resistance proteins predicted by the Cdvist web server tool and **b** phylogenetic relationship among the eight rice blast resistance protein trees generated using the neighbor-joining method by MEGA.7 software with bootstrap value 1000 replications

The CDvist Web with HMMER3 against Pfam 30.0 and Interpro scan server was performed to identify the domains of eight resistance proteins. NBS-LRR proteins are the plant disease resistance proteins, which share similar sequences and domains [23]. The results of the CDvist analysis revealed that the NB-ARC domain was found in all the eight resistance proteins and the LRR domain was identified in RP1, RP4, RP5, RP6, and RP7 proteins. RX-CC coiled-coil domain was identified in RP4, RP6, RP7, RP8 (Fig. 1a), and most of the NBS-LRR proteins contained some unknown domains, which were symbolized as X. Similarly, an Interpro scan server was used to predict the different domains: NB-ARC and LRR domain architectures were detected in all the eight resistance proteins. RX-CC domains are recognized in RP4, RP6, RP7, and RP8, and P-loop architecture was identified in RP1, RP2, RP3, RP4, RP6, and RP7 (Table 3).

**Table 3:** CDvist and Interpro Scan online tools performed for the identification of different domains (or motif analysis) for eight resistance proteins

Protein	CDvist		Interpro Scan	
	Domain	Region	Domain	Region
**RP1**	NB-ARC	124–406	NB-ARC	133–401
	LRR4	476–519	LRR	550–620
	LRR8	549–606	P-loop	137–377
**RP2**	_	_	NB-ARC	277–536
			P-loop	242–515
			LRR	577–968,
				978–1178
**RP3**	NB-ARC	644–925	NB-ARC	656–922
			P-loop	608–896
			LRR	1053–1450
**RP4**	RX-CC	99–217	RX-CC	98–216
	NB-ARC	254–535	NB-ARC	265–534
	LRR8	632–678	P-loop	255–506
	LRR4	689–736	LRR	619–937, 942–1056
**RP5**	NB-ARC	587–815	NB-ARC	598–811
	LRR4	939–975, 1197–1234	LRR	872–1068
				1069–1232
**RP6**	RX-CC	10–139	RX-CC	9–138
	NB-ARC	177–457	NB-ARC	187–455
	LRR8	555–604	P-loop	179–428
	LRR4	615–660, 791–826	LRR	516–864,
		905–952		895–1009
**RP7**	RX-CC	10–139	RX-CC	9–138
	NB-ARC	178–456	NB-ARC	187–454
	LRR8	552–603	P-loop	180–427
	LRR4	614–659,785–852	LRR	513–862, 894–1018
**RP8**	RX-CC	8–130	RX-CC	7–129
	NB-ARC	169–449	NB-ARC	183–447
			LRR	662–683, 723–745, 750–771

The amino acid sequences for eight resistance proteins aligned separately to identify their sequence diversity and phylogenetic relationships (Fig. 1b) revealed two distinct clades. RP5 and Pita branched out from RP4 and another cluster formed by RP3 and RP8 and they branched out from Pik2. In the other clade, RP1 and RP2 of resistant cultivar branched out from RP6 and RP7 and clustered out with reported blast resistance proteins.

### Protein modeling

**Fig. 2:**
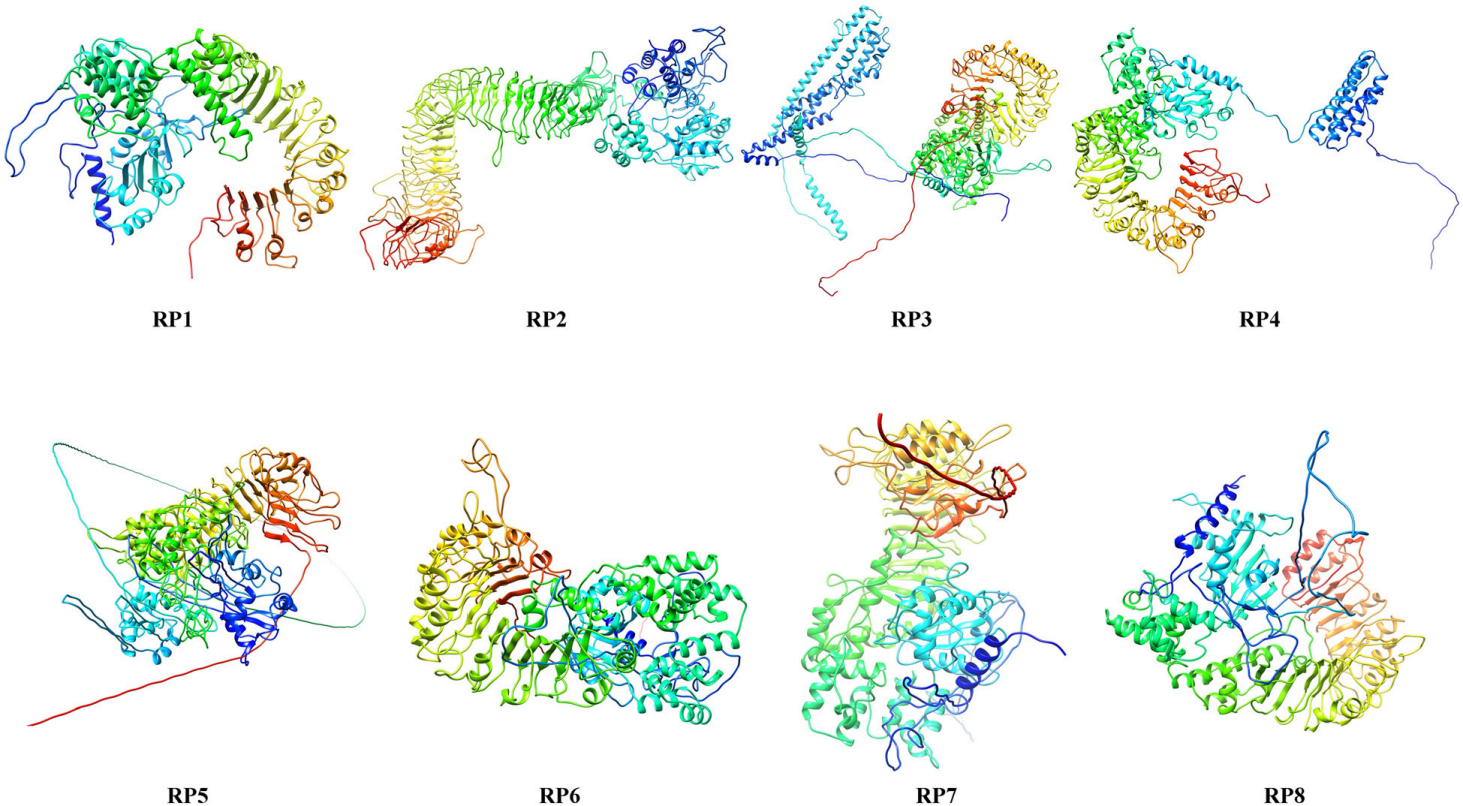
Three-dimensional structures of eight resistance protein RP1-RP8 models by RaptorX

SWISS-MODEL servers adopted to model the eight resistance proteins (Table 4) revealed that eight RPs share only 12.84–18.85% sequence identity and 26–65% query coverage with root-mean-square deviation (RMSD) of 1.74 Å–4.50 Å. Further, the top four templates were selected for each RP from the PSI-BLAST program to build the best target model by using Modeller 9.22 server. Template models were taken from Protein Data Bank. The best target models were selected based on the lowest DOPE (discrete optimized protein energy) for each RP in Modeller which led to our secondary structure predictions. These PDB files of eight resistance proteins were visualized by UCSF Chimera software (Fig. 2).

**Table 4:** Homology modeling of eight resistance proteins

Protein	Swiss Model				I-TASSER				PHYRE2		
	PDB ID	Identity	Query	RMSD	PDB ID	Identity	Query	RMSD	PDB ID	Identity	Query
		(%)	Coverage			(%)	Coverage			(%)	Coverage
RP1	4kxf.1.A	16.67	0.47	3.20 Å	3sfzA	0.11	0.84	2.76	c2a5yB	14.00	48.00
	4kxf.2.A	16.67	0.47	3.20 Å	1z6tB	0.13	0.50	2.63	c1vt4K	18.00	60.00
	4kxf.3.A	16.67	0.47	3.20 Å	3izaA	0.05	0.61	6.50	c1vt4J	18.00	60.00
	4kxf.4.A	16.67	0.47	3.20 Å	1vt4l	0.07	0.62	7.30	c3iz8E	18.00	60.00
RP2	4mn8.1.A	17.73	0.45	3.06 Å	3javA	0.09	0.97	1.70	c2a5yB	14.00	36.00
	4mn8.1.A	17.76	0.45	3.06 Å	5hb4B	0.04	0.47	9.25	clvt4P	14.00	44.00
	3jbl.1.A	15.66	0.42	4.50 Å	3opbA	0.04	0.37	6.85	clvt4L	14.00	44.00
	5gr8.1.A	16.64	0.42	2.59 Å	5fymA	0.03	0.44	9.68	clvt4K	14.00	44.00
RP3	4m9y.1.B	17.08	0.28	4.20 Å	6b5bA	0.11	0.78	2.43	c2a5yB	17.00	30.00
	2a5y.1.C	17.08	0.28	2.60 Å	4kxfK	0.08	0.45	5.21	c3iz8A	19.00	41.00
	2a5y.1.B	17.08	0.28	2.60 Å	1vt4l	0.04	0.49	8.96	c3iz8E	19.00	41.00
	3lqr.1.B	17.08	0.28	3.90 Å	3izaA	0.05	0.45	8.38	clvt4M	19.00	41.00
RP4	5gs0.1.A	16.06	0.26	3.28 Å	1z6tB	0.15	0.52	1.20	c2a5yB	15.00	42.00
	3ulv.1.A	16.33	0.26	3.52 Å	3sfzA	0.09	0.43	1.91	c1vt4N	15.00	51.00
	1ziw.1.A	16.14	0.26	2.10 Å	3vkgA	0.05	0.56	9.47	c3iz8F	15.00	51.00
	4lsa.1.A	17.82	0.27	3.30 Å	4ai6A	0.03	0.50	9.02	c3iz8A	15.00	51.00
RP5	4kxf.1.A	12.84	0.46	3.20 Å	6b5bA	0.08	0.86	2.01	c2a5yB	11.00	34.00
	4kxf.2.A	12.84	0.46	3.20 Å	4kxfK	0.06	0.51	5.38	c3iz8F	15.00	34.00
	4kxf.3.A	12.84	0.46	3.20 Å	1vt4l	0.04	0.55	8.42	c3iz8G	15.00	34.00
	4kxf.4.A	12.84	0.46	3.20 Å	3izaA	0.05	0.50	8.19	c3iz8A	15.00	34.00
RP6	3jbl.1.A	15.47	0.64	4.50 Å	6b5bA	0.12	0.86	2.06	c2a5yB	15.00	44.00
	5gs0.1.A	14.81	0.45	3.28 Å	4kxfK	0.08	0.55	4.93	c3iz8D	19.00	43.00
	4mn8.1.A	18.00	0.44	3.06 Å	1vt4l	0.07	0.59	8.34	c1vt4P	19.00	43.00
	4z0c.1.A	18.85	0.44	2.30 Å	5x6oC	0.04	0.58	8.30	c3iz8C	19.00	43.00
RP7	3jbl.1.A	14.33	0.65	4.50 Å	6b5bA	0.12	0.85	2.27	c2a5yB	15.00	44.00
	5ixo.1.A	16.89	0.42	1.74 Å	4kxfK	0.08	0.55	5.36	c3iz8G	17.00	43.00
	4lsx.1.A	17.54	0.43	3.30 Å	1vt4l	0.07	0.58	8.26	c3iz8F	17.00	43.00
	4lsa.1.A	17.54	0.43	2.50 Å	5x6oC	0.04	0.59	8.40	c1vt4O	17.00	43.00
RP8	3jbl.1.A	15.02	0.67	4.50 Å	4kxfK	0.13	0.82	2.47	c2a5yB	15.00	50.00
	4m9y.1.B	16.38	0.45	4.20 Å	6b5bA	0.05	0.62	5.46	c3iz8B	17.00	49.00
	2a5Y.1.C	16.38	0.45	2.60 Å	5irlA	0.08	0.58	5.85	c1vt4L	17.00	49.00
	2a5Y.1.B	16.38	0.45	2.60 Å	5h64A	0.05	0.67	8.46	3iz8F	17.00	49.00

### Secondary structure and function prediction

**Fig. 3:**
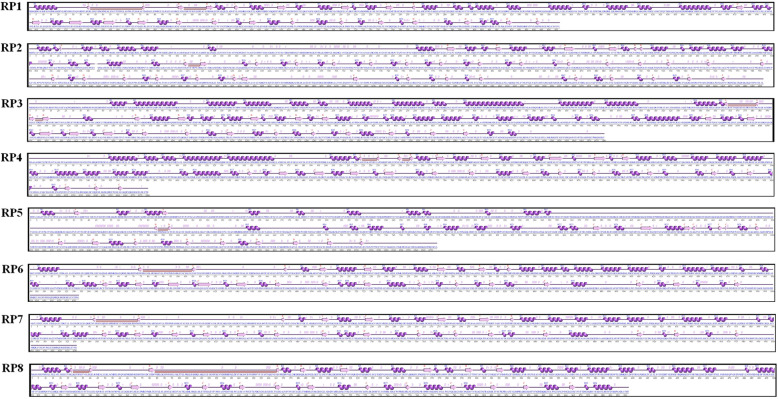
Secondary structures of eight resistance proteins by ProFunc server

The secondary structure predictions from the ProFunc server showed predominant alpha-helical coiled structures in all eight RPs (Fig. 3). In the RaptorX property prediction of eight RPs showed that 26–41% of residues are involved in the α-helices structure formation, 9–14% residues are arranged in β-strands, and 48–60% of the residues occur as coils. The solvent accesses of exposed, medium, and buried regions of eight RPs were found to be in the range of 26–31%, 38–48%, and 25–30% in RaptorX prediction function.

**Fig. 4:**
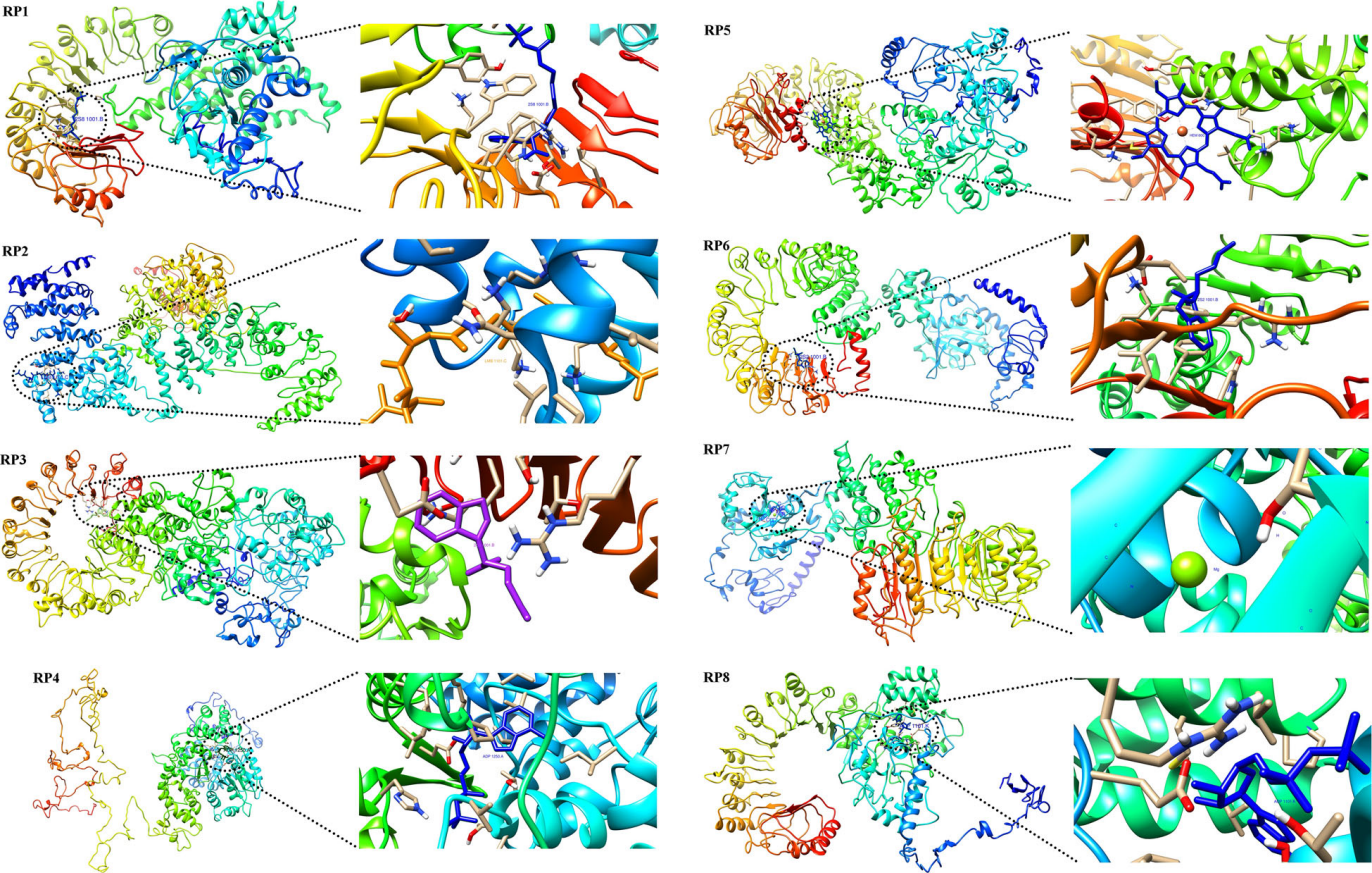
Binding sites of RP1, RP2, RP3, RP4, RP5, RP6, RP7, and RP8 resistance proteins by using the COACH server based on the I-TASSER structure prediction

The COACH server based on the I-TASSER structure prediction used to predict the active sites of the eight resistance proteins (Fig. 4) revealed that the ADP binding site of RP1 was mainly composed of the amino acid numbers V115, R117, T118, F119, R121, G145, G146, G148, K149, T150, and T151. The important active site residues having binding activity for LMB (Leptomycin B) of RP2 were C181, H188, K192, P195, K198, and R201. The amino acid residues with a binding site for 2S2-(2S)-2-(1H-INDOL-3-YL) hexanoic acid were found to be D1366, R1368, T1388, V1389, S1390, R1427, K1429, I1430, D1465, I1466, and A1467 in RP3. The amino acid residues of RP4 with binding sites for ADP were P247, L249, V250, G251, I254, G282, G283, V284, G285, K286, T287, T288, P446, L447, K450, E556, and H570 [24]. L1254 and D1258 amino acid residues were involved in the binding site for HEM in RP5. The amino acid residues V849, K851, D911, L912, V913, K934, F936, I937, F977, V978, and N979 were the binding site residues for 2S2 - (2S)-2-(1H-INDOL-3-YL) hexanoic acid in RP6. The ligand-binding site residues for RP7 were T208 and L279 involved in the ligand MG binding site. The ligand-binding site for RP8 was L136, E138, V153, Y156, A157, N158, G189, L190, G191, K192, T193, T194, L321, L329, P360, L361, and M364 involved in the ADP binding site (Table 5) [25].

**Table 5:** Ligand binding site predictions of eight resistance proteins

Protein	Rank	C-score	Cluster size	PDB hit	Ligand name	Ligand binding site residues
RP1	1	0.09	4	3sfzA	ADP	**115, 117–119, 121, 145, 146, 148-151**
	2	0.07	4	4m9sC	PEPTIDE	**360, 363, 364, 371, 372, 375, 455, 456, 459**
	3	0.05	2	5aorB	MG	**150, 216**
	4	0.04	2	3shfA	GBL	**378, 379, 453, 460**
	5	0.02	1	5hhjA	GLY	**271, 274**
RP2	1	0.04	3	4hb4C	LMB	**181, 188, 192, 195, 198, 201**
	2	0.04	3	3an2E	Nuc. Acid	**828, 829, 830**
	3	0.03	2	2z4rA	MG	**295, 365, 366**
	4	0.03	2	2a5yB	ATP	**255, 258, 290–296, 397, 425, 429, 457, 458**
	5	0.03	2	4hazC	LBF	**182, 189, 192, 201, 204**
RP3	1	0.09	6	2a5yB	2S2	**1366, 1368, 1388–1390, 1427, 1429, 1430, 1465–1467**
	2	0.09	6	3c6oB	ATP	**621, 624, 671–677, 779, 806, 810, 837, 838, 841, 868**
	3	0.03	2	5d0fA	MTT	**939, 942, 943, 946**
	4	0.02	1	2a5yC	MG	**676, 748**
	5	0.02	1	N/A	N/A	**884, 939**
RP4	1	0.21	7	3sfzA	ADP	**247, 249–251, 254, 282–288, 446, 447, 450, 556, 570**
	2	0.05	2	4m9zA	MG	**287, 359, 360, 386**
	3	0.05	2	2qbyA	MG	**287, 359, 447**
	4	0.03	1	N/A	N/A	**844, 866, 870, 872, 874, 926, 933, 935, 941, 945, 947**
	5	0.02	1	N/A	N/A	**621, 625, 628, 665, 669**
RP5	1	0.07	5	1m7sA	HEM	**1254, 1258**
	2	0.04	3	3cr3A	MG	**638, 639**
	3	0.03	2	4rkuG	CLA	**773, 776**
	4	0.03	2	4hzcG	MG	**760, 762**
	5	0.02	1	N/A	N/A	**1142, 1145, 1154, 1156, 1160, 1164–1168, 1173, 1176**
RP6	1	0.08	4	3c6oB	2S2	**849, 851, 911–913, 934, 936, 937, 977, 978, 979**
	2	0.06	3	2p1nB	CFA	**728, 730, 764, 765, 766, 789, 790, 812–814**
	3	0.03	1	1vt4l	MG	**208, 279**
	4	0.02	1	1koiA	NO	**568, 571**
	5	0.02	1	3w3A	L07	**704, 733, 769**
RP7	1	0.10	5	1vt4I	MG	**208, 279**
	2	0.08	5	2a5yB	ATP	**168, 171, 203–209, 309, 336, 340, 367, 368, 371, 399,**
	3	0.06	4	3c6oB	2S2	**811, 813, 848–850, 909, 911, 936, 937, 938**
	4	0.05	3	3ogkB	OGK	**727, 763–765, 788, 790, 813, 848, 913, 935**
	5	0.05	3	2p1pB	IAC	**911, 936–938, 977, 978, 997, 998, 999, 1029**
RP8	1	0.23	11	4kxfK	ADP	**136, 138, 153, 156–158, 189–194, 321, 329, 360, 361, 364**
	2	0.08	4	3u60E	MG	**193, 270, 271, 361**
	3	0.04	2	3t6qA	MAN	**616, 641**
	4	0.02	1	3w3nB	RX8	**571, 573, 574, 597, 599, 623**
	5	0.02	1	3b2dA	MAN	**662, 687**

PROFUNC predicts the cellular component, biological process, and biochemical function of eight resistance proteins, and the results are depicted in Table 6. The PROFUNC predicts the probable functions based on the 3D structure of the target protein [26]. The eight resistance proteins showed scores in the cellular, biological, and biochemical functions. The cellular component scores ranged from 1.68–38.14, biological process ranged between 3.73–40.39, and biochemical function were in the range of 13.61–49.87.

**Table 6:** Gene Ontology of eight resistance proteins

Functions	RP1	RP2	RP3	RP4	RP5	RP6	RP7	RP8
	Score							
**Cellular component**								
Cell	22.79	16.55	7.89	22.18	4.83	19.57	38.14	10.12
Cell Part	22.79	16.55	7.89	22.18	4.83	19.57	38.14	10.12
Membrane	17.87	–	–	17.53	3.73	12.30	25.02	5.43
Integral to membrane	9.17	–	–	–	1.68	–	17.30	–
Membrane part	–	–	–	8.29	–	–	–	–
Intracellular	–	9.54	4.08	–	–	–	–	–
Intracellular part	–	9.54	4.08	–	–	–	–	–
Plasma membrane	–	–	–	–	–	–	–	–
Cytoplasm	–	–	–	–	–	–	–	4.90
**Biological process**								
Cellular Process	33.37	18.60	12.35	32.50	4.83	22.17	40.39	10.94
Biological regulation	27.08	14.82	7.69	25.67	4.83	16.53	34.00	10.07
Regulation of biological process	26.49	14.82	7.69	25.67	4.83	–	33.44	10.07
Regulation of cellular process	25.57	14.05	–	25.67	–	–	33.44	10.07
Cellular metabolic process	–	–	6.26	–	–	–	–	–
Signal transduction	–	–	–	–	3.73	–	–	–
Response to stimulus	–	–	–	–	–	18.37	–	–
Cell communication	–	–	–	–	–	17.59	–	–
**Biochemical function**								
Binding	40.31	26.66	16.61	45.84	19.76	35.06	49.87	34.45
Nucleotide Binding	26.25	24.31	13.61	29.04	14.26	30.82	29.74	29.11
Purine nucleotide binding	26.25	24.31	13.61	29.04	14.26	30.82	29.74	29.11
Purine ribonucleotide binding	–	–	–	29.04	–	30.82	29.74	–
ADP binding	–	–	–	–	14.26	–	–	–
Adenyl nucleotide binding	26.25	24.31	13.61	–	–	–	–	29.11

### SuperPose

The root means square deviation (RMSD) that measures the distance between corresponding residues and accurate models should have < 2.0 Å value [27]. RMSD calculates how much the resistance protein deviates from each other. The eight RPs superimposed each other separately using all the permutations and combinations (Table 7) showed that RP5 was superimposed with RP8 with the least RMSD value (1.2 Å). The RP1, RP2, RP3, RP4, RP6, and RP7 were having identical three-dimensional structures and having an RMSD value ranging between 1.45 and 2.22 Å. The superimposed structures of all eight-resistance protein models indicate that the overall conformations are very similar except RP5.

**Table 7:** Superpose predictions of eight resistance proteins

Gene/RMSD	RP1	RP2	RP3	RP4	RP5	RP6	RP7	RP8
**RP1**	–	2.01	2.22	1.65	82.15	1.45	1.56	23.22
**RP2**	2.01	–	41.79	1.96	83.68	4.23	4.33	2.68
**RP3**	2.22	41.79	–	1.83	83.80	1.39	1.43	1.80
**RP4**	1.65	1.96	1.83	–	87.09	1.69	2.05	2.06
**RP5**	82.15	83.68	83.80	87.09	–	85.11	82.23	1.20
**RP6**	1.45	4.23	1.39	1.69	85.11	–	1.78	2.35
**RP7**	1.56	4.33	1.43	2.05	82.23	1.78	–	1.96
**RP8**	23.22	2.68	1.80	2.06	1.20	2.35	1.96	–

### Structure validation

**Fig. 5:**
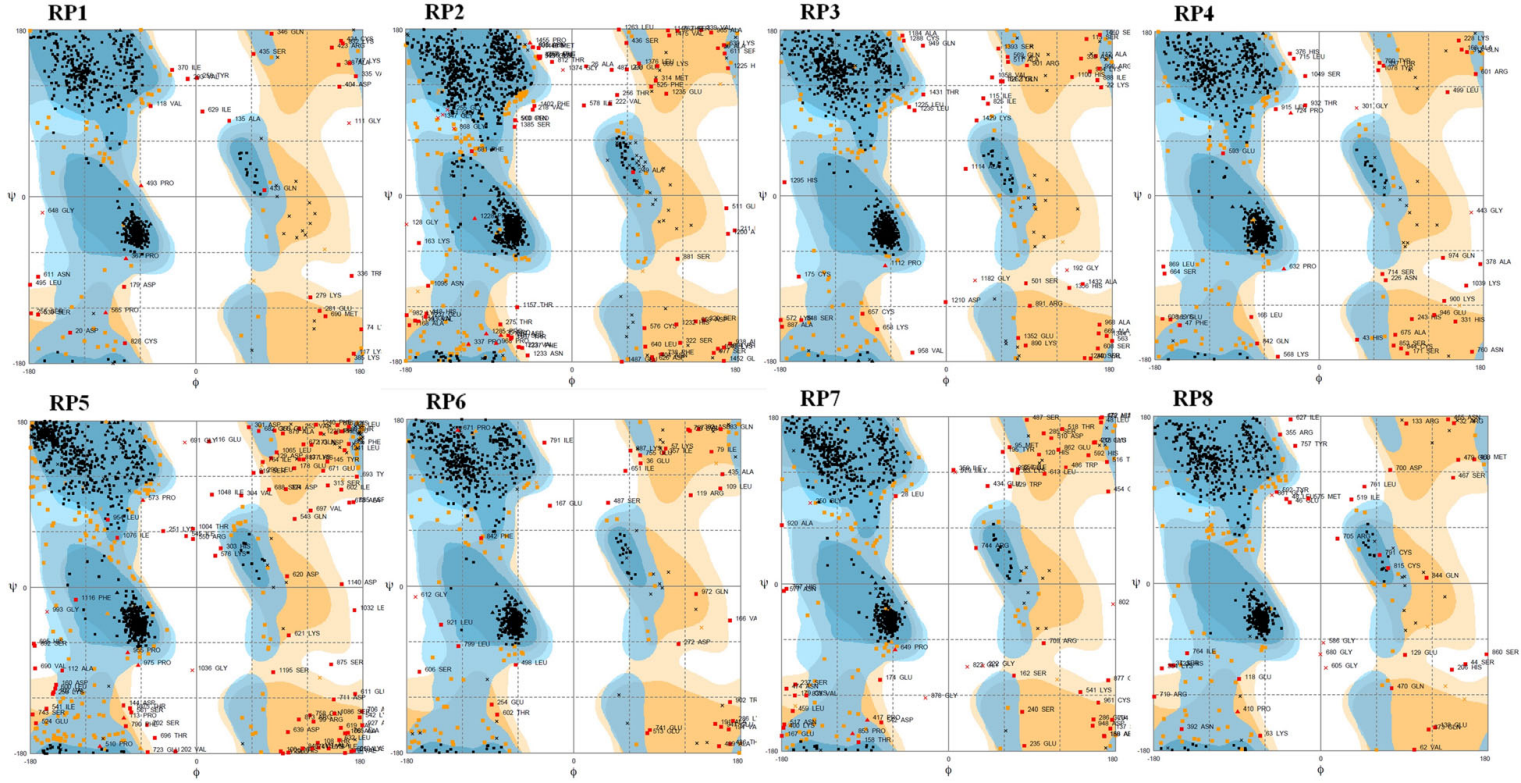
Ramachandran plot statistics of eight resistance proteins

Ramachandran plot showed the distribution of φ and ψ angle in the eight resistance protein models within the limits (Fig. 5). Ramachandran plot statistics displayed that 647 amino acid residues (83.1%) are in the favored region, 99 amino acid residues (12.7%) are in the additional allowed region, and 24 amino acid residues (3.1%) are in the generously allowed region, while only nine amino acid residues (1.2%) are in the disallowed region in RP1. RP2 showed that 1323 amino acid residues (98.4%) are in the allowed region and 21 amino acid residues (1.6%) are in the disallowed region. RP3 displayed 1223 amino acid residues (98.3%) are in the allowed region and 21 (1.7%) in the disallowed region. RP4 showed 966 amino acid residues (98.2%) are in the allowed and 17 amino acid residues (1.7%) in the disallowed region. RP5 showed 1101 amino acid residues (94.9%) is allowed and 58 amino acid residues (5.8%) in the disallowed region. RP6 showed 908 amino acid residues (98.0%) in the allowed region and 17 amino acid residues (1.8%) in the disallowed region. RP7 identified 905 amino acid residues (97.4%) is allowed and 24 amino acid residues (2.6%) in the disallowed region. RP showed 804 amino acid residues (98.3%) in the allowed and14 amino acid residue (1.7%) in the disallowed region. Ramachandran plot of eight resistance proteins confirmed that the model structures are following dihedral angles of Ramachandran plot occupied favorable positions [12].

## Discussion

In rice plants, many resistance genes are mostly polymorphic [28] which are involved to initiate the cascade signaling to trigger the defense response in rice plants. Understanding the differential gene expression of resistance genes and their action may give new insights to study the differential transcriptional regulation in rice plant and model the blast resistance protein structures which are expressed during *M. oryzae* infection.

Transcriptomic studies carried out during the present investigation revealed some common transcripts for both BR2655 and HR12. We also observed many of the transcripts are unique to the resistant cultivar, viz., BR2655, and susceptible cultivar HR12 separately. A similar difference in the transcriptomic profiles was observed in Italian rice varieties Gigante Vercelli and Vialone Nano cultivars of rice [29]. Many researchers have carried out previously transcriptomic studies in rice cultivars inoculated with different pathogens [30, 31]. Many such studies are carried out in different plants like tomato, banana, and maize [32, 33].

We observed the difference in the response of pathogenesis and gene expression profiling in BR2655 and HR12 rice cultivars during infection by *M. oryzae.* When the transcripts of cultivars are analyzed, it is important to analyze different categories of transcripts expressed, viz., only in resistant, both in resistant and susceptible and only in susceptible cultivars. Accordingly, we identified the R genes which are expressed exclusively in the resistance cultivar BR2655 which may play a decisive role in conferring resistance to the plants. The *R* genes which are expressed in both may be responsible for initiating the defensive mechanism. The *r* genes which are expressed exclusively in susceptible cultivars may not be effective in overcoming a specific pathogen like *M. oryzae* due to change in the cascade of multiple signaling pathways resulting in the virulence of a given pathogen.

In the present study, we thus have shortlisted eight blast resistance transcripts, two each exclusively from BR2655 and HR12 and four common to both the rice cultivars, and their protein sequences were retrieved for further computational analysis and characterization. Resistance proteins are broadly classified into eight groups based on their different conserved domain organization and secondary structures. These resistance proteins are the key players in the plant defense signaling mechanisms [4]. All the shortlisted resistance proteins bearing NBS were observed, and physiochemical properties of all the RP proteins were analyzed in the current study.

### Motifs and phylogenetic tree

The eight resistance proteins encode nucleotide-binding site leucine-rich repeats (NBS-LRR) involved in the defense mechanism which share similar sequences and domains [23]. NBS-LRR domains investigated during the current study and most of the domains reported by earlier workers contained some unknown domains, which were symbolized as X [34].. Coiled-coil amino acid sequences of resistance proteins are involved in the signal transduction in many cell processes [35]. NB-ARC and LRR domain architectures were detected in all the eight resistance proteins. These domains play an important role in plant resistance gene inactivation of downstream effectors [36]. The conserved amino acid residues of RP1 was involved in the binding of ADP and reported to be involved in signal transduction [37]. RP2 involves in the binding activity for LMB (Leptomycin B), and RP4 conserved amino acid residues were involved in the binding site for 2S2 - (2S)-2-(1H-INDOL-3-YL) hexanoic acid, having binding sites for ADP [37] and RP8 conserved amino acid residues in the binding site residues for 2S2-(2S)-2-(1H- INDOL-3-YL) hexanoic acid, which acts as ADP binding site [38]. The results revealed that these eight resistance proteins might involve in the transmembrane transport of metal ions.

A phylogenetic tree generated based on the amino acid sequences of these 8 resistance proteins is divided into two groups. A similar analysis was reported for resistance proteins in verticillium wilt-resistant cotton plants [32]. The eight resistant proteins are having a structural and functional relationship with each other indicating that these proteins are well conserved and evolved in the same family of their evolutionary history. Similar studies were also reported in Arabidopsis during pathogen attack *Pseudomonas syringae* [39].

The physico-chemical properties of eight resistance proteins and their amino acid composition revealed the existence of high-frequency leucine-rich repeats. They are involved in hydrophobic interactions and conformational stability of the RP proteins [40]. The aliphatic index of hypothetical eight RPs revealed that they are stable even at high temperatures. These RPs have hydrophilic amino acids which are capable of interacting with surrounding water molecules intracellularly [41].

### Homology modeling

SWISS-Model and Modeller servers were used to build the three-dimensional structures of eight RPs. The templates showed similarity or identity for the eight resistance proteins, and these template models are associated with cell death protein 4 (PDB ID: 3lqr.1.A, PDB ID: 2a5y.1.B), which are localized to the nucleus in proliferating cells [42].

Recently, the cryo-electron microscopy structure of NBS-LRR, such as the wheel-like pentameric ZAR1 resistosome, has been revealed in *Arbidopsis thaliana* [43]. Similar template structures were used for modeling in the current study, and it helped us to decipher the binding site variations that may occur in different resistance proteins. RP2 was found to be similar to LRR receptor-like serine/threonine-protein kinase FLS2 (PDB ID: 4mn8.1.A) which functions as a pattern recognition receptor [37]. RP4 resembles Toll-like receptor 3 (PDB ID: 5gs0.1.A) which functions as pathogen recognition and activation of innate immunity protein [44], RP5 NLR family CARD Domain-containing Protein 4 (PDB ID: 4kxf.3.A, PDB ID: 3jbl.1.A, PDB ID: 3jbl.1.A, PDB ID: 3jbl.1.A), and LR family CARD Domain-containing Protein 4 (PDB ID: 3jbl.1.A) which indirectly senses specific proteins from pathogenic bacteria and fungi [45, 46]. This paper sheds light on the modeling of eight hypothetical resistance proteins showing homology to the template models which are mainly involved in defense mechanisms. Similar protein modeling carried out and reported on the orthologue of *Pi54* designated as *Pi54of* from *Oryza officinalis* was studied and modeled [8, 47].

### Protein structure, function, and validation

The secondary structure predicted for the eight-resistance protein by using RaptorX showed that residues are involved in the formation of α-helix, β-sheet, and coils structures. Profunc predicts the probable functions based on the 3D structure of the target protein [26]. SuperPose detects the root means square deviation (RMSD) that measures the distance between corresponding residues and accurate models should have < 2.0 Å value [27]. Ramachandran plot of eight resistance proteins confirmed that the modeled structures are following the dihedral angles of the Ramachandran plot and occupied favorable positions (Fig. 5) [2]. Further work is needed to understand the difference between resistance proteins of resistant and susceptible rice cultivars. This can be understood once the structure of effector molecules expressed by the *Avr* genes of the pathogen is available. Hence, there is a need to elucidate the effector molecules to understand the interaction of resistance proteins and effector proteins.

Disease resistance in plants is more often regulated by a gene-for-gene mechanism in which Avr proteins encoded by pathogens are particularly detected by plant disease R proteins directly or indirectly. Avr proteins trigger defense response elements by changing the membrane ion flux, irreversible plasma membrane damage, production of extracellular reactive oxygen intermediates, and alter in gene expression [48]. The protein-protein interaction of Avr and R proteins becomes evident by the ability of a host to detect pathogen effectors. However, there are relatively few reports on direct interactions between Avr and R proteins [49–52]. Avr proteins presumably enhance the virulence factors by hindering the innate immune systems of host plants in the absence of recognition by R proteins [53, 54]. Few *Avr* genes are identified and their protein interactions with resistance protein are yet to be structurally characterized [6].

The present study was aimed to identify the transcripts involved in rice blast resistance in resistant BR2655 and susceptible HR12 cultivars and model the three-dimensional structures, function predictions, conserved motifs, and validations of NBS-LRR of eight hypothetical resistance proteins (RP1, RP2, RP3, RP4, R5, RP6, RP7, and RP8) using computational tools. No previous studies are found on this NBS-LRR of eight resistance proteins. Hence, we have modeled the eight resistance proteins, which are found to be stable, with well-defined compact reliable three-dimensional structures, by using highly reputed computational tools. The eight resistance proteins were modeled using SWISS-MODEL, I-TASSER, and RaptorX server tools. The secondary structure predicted by RaptorX and ProFunc displayed the presence of α-helix, β-strands, and random coils. ProFunc, Motif, SuperPose, and Ramachandran plot servers were used to predict the structure and function of eight resistance proteins. These eight resistance proteins will function as a valuable resource for studying the intricate details of the plant defense mechanism.

## Conclusions

In silico studies provide an opportunity to accomplish the modeling and analysis of resistance proteins by employing various modeling applications. In the current study, blast resistance transcripts expressed were shortlisted by transcriptomic profiling. Protein sequences of expressed transcripts were selected to determine the physicochemical properties and structures of resistance proteins using in silico techniques. Primary structure analysis revealed that all the resistant and susceptible encoded resistance proteins are rich in leucine residues which seem to correlate with the reported resistance proteins. The secondary structure analysis confirmed that in all the eight sequences, alpha helix dominated, followed by beta turns and then coils. Three-dimensional structure predictions were analyzed by different homology servers, viz., Swiss model and Modeller 9.22. The model structures were validated by a protein structure checking tool called Rampage. The in silico modeled eight resistance proteins are promising candidates for providing insights into domain structures. We hope that further studies with the structure of these resistance proteins and their interactions will provide a better insight into the precise molecular mechanism involved in plant defense.

## Supplementary Information

**Additional file 1:** Supplementary Data 1, 2, 3 and 4. Supplementary Figures 1 and 2. Tables 1-4.

## Data Availability

All data generated during this study are included in this manuscript [and its supplementary information].

## Abbreviations

NBS-LRR: Nucleotide-binding site-leucine-rich repeat
RP: Resistance protein
CC: Coiled-coil motif
TIR: Toll-interleukin-1-receptor
DEG: Differentially expressed genes
RMSD: Root means square deviation
PDB: Protein data bank
