# Supplementary material for: In silico modelling and characterization of eight blast resistance proteins in resistant and susceptible rice cultivars
Source: J Genet Eng Biotechnol. 2020 Nov 25;18:75. doi: 10.1186/s43141-020-00076-0 (PMC7688789; doi:10.1186/s43141-020-00076-0)
Supplement: Supplementary file 1 — Additional file 1: Supplementary Data 1, 2, 3 and 4. Supplementary Figures 1 and 2. Tables 1-4. [file 43141_2020_76_MOESM1_ESM.docx]

**Supplementary Document**

**Supplementary Figure 1:** Flow chart of work carried out


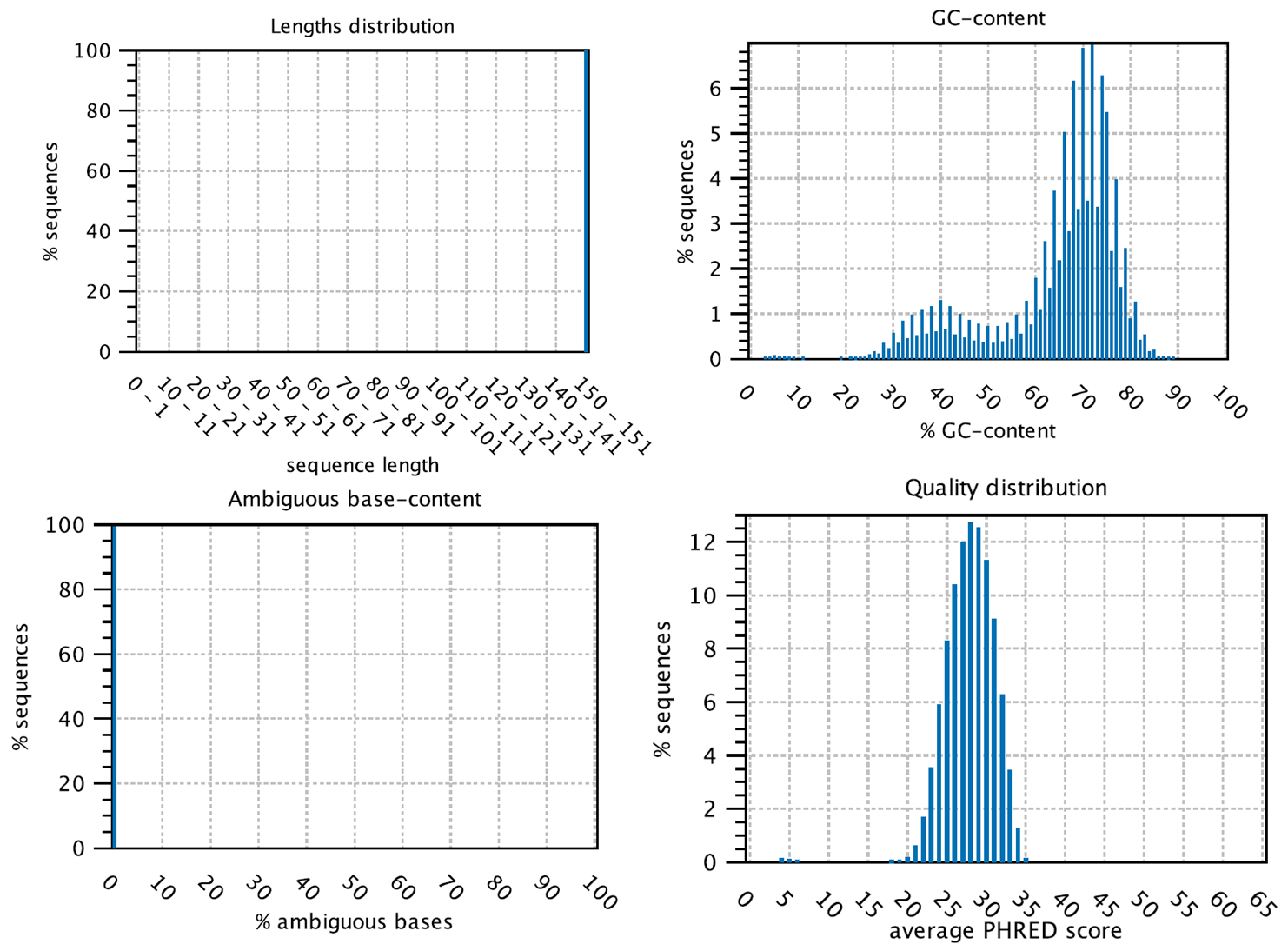

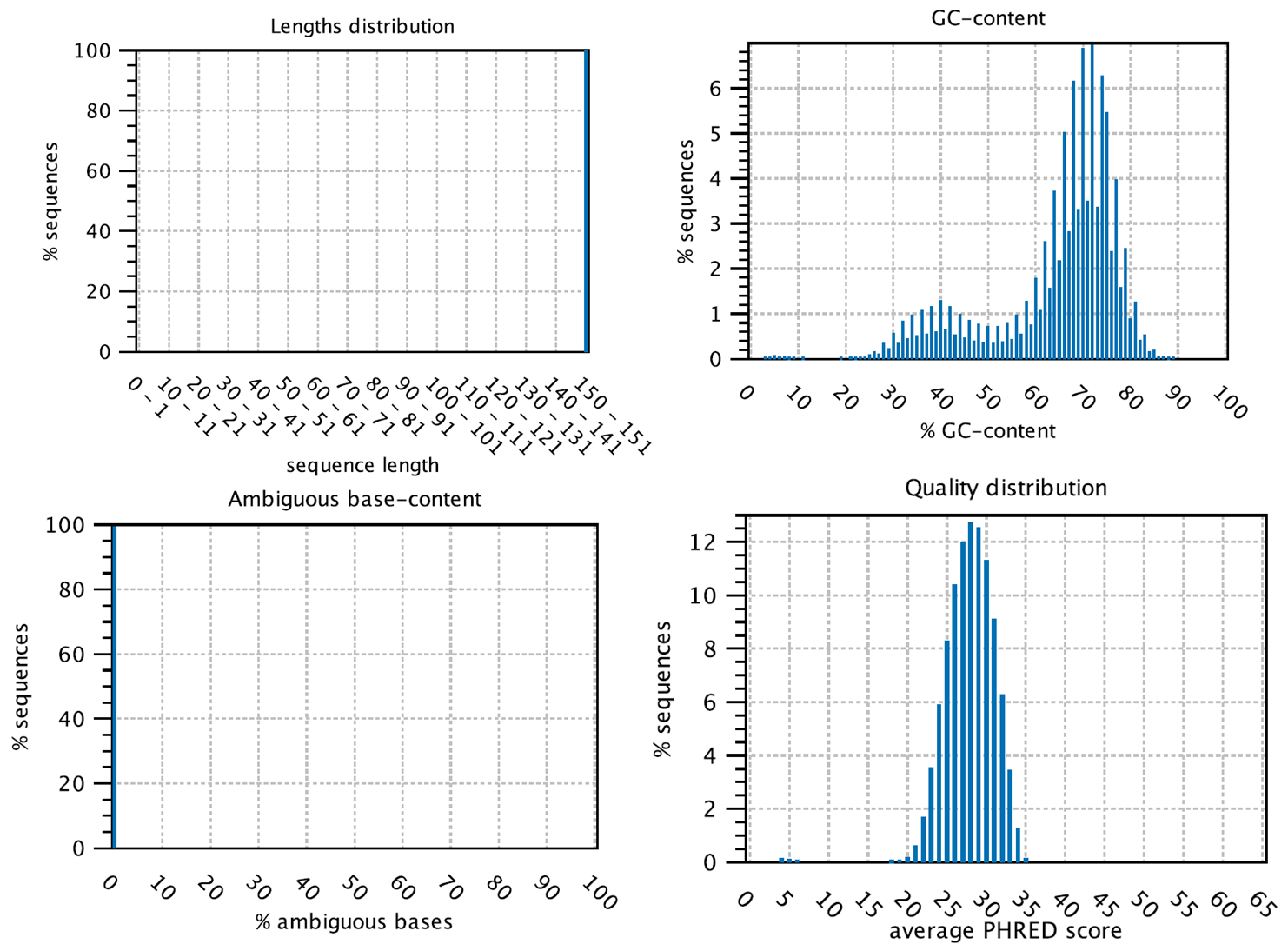


**B**

**A**

**Supplementary Figure 2:** Quality of sequences obtained for four samples (BRC, BRIN, HRC and HRIN) by using CGWB A. Sequence length and B. PHRED Score

**Supplementary Table 1:** Genes taken for the structure determination from RGAP

| **Sl. no** |  | **Cultivars** | | | |  |
| --- | --- | --- | --- | --- | --- | --- |
|  |  | **BR2655** | | **HR12** | |  |
|  |  | **Fold change** | **P-valve** | **Fold change** | **P-value** |  |
| **1** | LOC_Os01g72390 | 21 | 0.002569 | - | - | NBS type disease resistance protein, ... [v7] |
| **2** | LOC_Os07g04900 | 10.5 | 0.001194 | - | - | NBS-LRR disease resistance protein, p... [v7] |
| **3** | LOC_Os12g09240 | 8.5 | 0.003551 | 7.5 | 0.001616 | NBS-LRR disease resistance protein, p... [v7] |
| **4** | LOC_Os09g14490 | 3.67 | 0.008827 | 3.91 | 0.002075373 | TIR-NBS type disease resistance prote... [v7] |
| **5** | LOC_Os11g44960 | 3.45 | 0.013905 | 4.58 | 1.49315E-07 | NBS-LRR disease resistance protein, p... [v7] |
| **6** | LOC_Os11g13940 | 6 | 0.014305 | 6.25 | 0.005825444 | NBS-LRR disease resistance protein, p... [v7] |
| **7** | LOC_Os11g38580 | - | - | 17.5 | 5.79E-08 | NBS-LRR type disease resistance prote... [v7] |
| **8** | LOC_Os11g45180 | - | - | 35 | 6.15047E-05 | NBS-LRR disease resistance protein, p... [v7] |

**Supplementary Table 2:** List of upregulated genes shortlisted by search of Resistance genes by the key word ‘LRR’ in HR12, Resistance in HR12

| **Sl.no.** | **Locus ID** | **Fold change** | **P-valve** | **Description** |
| --- | --- | --- | --- | --- |
| **LRR in HR12** | | | | |
| 1 | LOC_Os11g45180 | 35 | 6.15E-05 | NBS-LRR disease resistance protein, p... [v7] |
| 2 | LOC_Os12g33210 | 23 | 0.001496 | OsFBL59 - F-box domain and LRR contai... [v7] |
| 3 | LOC_Os03g56250 | 21.625 | 0 | LRR receptor-like protein kinase, put... [v7] |
| 4 | LOC_Os11g38580 | 17.5 | 5.79E-08 | NBS-LRR type disease resistance prote... [v7] |
| 5 | LOC_Os12g17340 | 15 | 0.013328 | CC-NBS-LRR resistance protein MLA13, ... [v7] |
| 6 | LOC_Os12g10400 | 12 | 0.030984 | NBS-LRR type resistance protein, puta... [v7] |
| 7 | LOC_Os05g45870 | 11 | 0.000407 | OsFBL25 - F-box domain and LRR contai... [v7] |
| 8 | LOC_Os04g53160 | 10.2 | 1.38E-05 | NBS-LRR disease resistance protein, p... [v7] |
| 9 | LOC_Os12g09240 | 7.5 | 0.001616 | NBS-LRR disease resistance protein, p... [v7] |
| 10 | LOC_Os06g06380 | 6.666667 | 0.012193 | NBS-LRR type disease resistance prote... [v7] |
| 11 | LOC_Os11g13940 | 6.25 | 0.005825 | NBS-LRR disease resistance protein, p... [v7] |
| 12 | LOC_Os11g44960 | 4.583333 | 1.49E-07 | NBS-LRR disease resistance protein, p... [v7] |
| 13 | LOC_Os11g11810 | 4.25 | 0.044862 | NBS-LRR disease resistance protein, p... [v7] |
| 14 | LOC_Os06g03500 | 4 | 0.033894 | NBS-LRR disease resistance protein, p... [v7] |
| 15 | LOC_Os02g38392 | 3.333333 | 0.002142 | NBS-LRR disease resistance protein, p... [v7] |
| 16 | LOC_Os04g43440 | 3.173913 | 0.000308 | NB-ARC/LRR disease resistance protein... [v7] |
| 17 | LOC_Os11g39160 | 3 | 0.033894 | NBS-LRR disease resistance protein, p... [v7] |
| **RESISTANCE in HR12** | | | | |
| 1 | LOC_Os11g45180 | 35 | 6.15E-05 | NBS-LRR disease resistance protein, p... [v7] |
| 2 | LOC_Os11g41210 | 30 | 0.00023 | disease resistance protein RPM1, puta... [v7] |
| 3 | LOC_Os11g38580 | 17.5 | 5.79E-08 | NBS-LRR type disease resistance prote... [v7] |
| 4 | LOC_Os12g17340 | 15 | 0.013328 | CC-NBS-LRR resistance protein MLA13, ... [v7] |
| 5 | LOC_Os11g11920 | 13 | 0.023342 | resistance protein, putative, expressed [v7] |
| 6 | LOC_Os12g11370 | 12.44444 | 3.56E-11 | verticillium wilt disease resistance ... [v7] |
| 7 | LOC_Os11g41540 | 12 | 0.030984 | disease resistance RPP8-like protein ... [v7] |
| 8 | LOC_Os12g10400 | 12 | 0.030984 | NBS-LRR type resistance protein, puta... [v7] |
| 9 | LOC_Os10g07400 | 10.33333 | 0.000685 | disease resistance RPP13-like protein... [v7] |
| 10 | LOC_Os04g53160 | 10.2 | 1.38E-05 | NBS-LRR disease resistance protein, p... [v7] |
| 11 | LOC_Os02g27710 | 9.5 | 0.008712 | disease resistance protein, putative,... [v7] |
| 12 | LOC_Os01g42410 | 9.307692 | 4.19E-11 | pleiotropic drug resistance protein, ... [v7] |
| 13 | LOC_Os01g42370 | 9 | 0.011412 | pleiotropic drug resistance protein, ... [v7] |
| 14 | LOC_Os02g35210 | 9 | 0.011412 | resistance protein, putative, expressed [v7] |
| 15 | LOC_Os01g31870 | 8.833333 | 9.42E-10 | natural resistance-associated macroph... [v7] |
| 16 | LOC_Os12g12130 | 8 | 5.31E-05 | verticillium wilt disease resistance ... [v7] |
| 17 | LOC_Os12g32814 | 8 | 0.000967 | pleiotropic drug resistance protein 3... [v7] |
| 18 | LOC_Os12g09240 | 7.5 | 0.001616 | NBS-LRR disease resistance protein, p... [v7] |
| 19 | LOC_Os06g47800 | 6.666667 | 0.000393 | disease resistance protein RGA3, puta... [v7] |
| 20 | LOC_Os06g06380 | 6.666667 | 0.012193 | NBS-LRR type disease resistance prote... [v7] |
| 21 | LOC_Os11g13940 | 6.25 | 0.005825 | NBS-LRR disease resistance protein, p... [v7] |
| 22 | LOC_Os09g14100 | 6 | 0.020637 | disease resistance protein RPS2, puta... [v7] |
| 23 | LOC_Os09g19330 | 5 | 0.0455 | stripe rust resistance protein Yr10, ... [v7] |
| 24 | LOC_Os08g29570 | 4.714286 | 4.78E-07 | pleiotropic drug resistance protein 3... [v7] |
| 25 | LOC_Os11g44960 | 4.583333 | 1.49E-07 | NBS-LRR disease resistance protein, p... [v7] |
| 26 | LOC_Os11g11810 | 4.25 | 0.044862 | NBS-LRR disease resistance protein, p... [v7] |
| 27 | LOC_Os11g12330 | 4.166667 | 1.39E-06 | disease resistance protein RPM1, puta... [v7] |
| 28 | LOC_Os04g49890 | 4.064516 | 8.26E-08 | multidrug resistance-associated prote... [v7] |
| 29 | LOC_Os06g03500 | 4 | 0.033894 | NBS-LRR disease resistance protein, p... [v7] |
| 30 | LOC_Os09g14490 | 3.909091 | 0.002075 | TIR-NBS type disease resistance prote... [v7] |
| 31 | LOC_Os12g39620 | 3.636364 | 9.3E-09 | disease resistance protein, putative,... [v7] |
| 32 | LOC_Os11g11960 | 3.5 | 4.45E-05 | disease resistance protein RPM1, puta... [v7] |
| 33 | LOC_Os02g38392 | 3.333333 | 0.002142 | NBS-LRR disease resistance protein, p... [v7] |
| 34 | LOC_Os11g12340 | 3.190476 | 0.000525 | disease resistance protein RPM1, puta... [v7] |
| 35 | LOC_Os04g43440 | 3.173913 | 0.000308 | NB-ARC/LRR disease resistance protein... [v7] |
| 36 | LOC_Os10g04342 | 3 | 0.004677 | stripe rust resistance protein Yr10, ... [v7] |
| 37 | LOC_Os11g39160 | 3 | 0.033894 | NBS-LRR disease resistance protein, p... [v7] |
| 38 | LOC_Os12g39180 | 3 | 0.033894 | natural resistance-associated macroph... [v7] |

**Supplementary Table 3:** List of upregulated Genes shortlisted by search of Resistance genes by the key word ‘LRR’ in BR2655 and ‘Resistance’ in BR2655

| **Sl.no.** | **Locus ID** | **Fold change** | **P-valve** | **Description** |
| --- | --- | --- | --- | --- |
| **LRR in BR2655** | | | | |
| 1 | LOC_Os07g04900 | 10.5 | 0.001194 | NBS-LRR disease resistance protein, p... [v7] |
| 2 | LOC_Os12g34220 | 9.5 | 0.002055 | OsFBL61 - F-box domain and LRR contai... [v7] |
| 3 | LOC_Os12g32670 | 9.5 | 0.008712 | CC-NBS-LRR resistance protein, putati... [v7] |
| 4 | LOC_Os04g53496 | 8.571429 | 4.68E-06 | NBS-LRR disease resistance protein, p... [v7] |
| 5 | LOC_Os05g49980 | 8.5 | 0.003551 | OsFBL26 - F-box domain and LRR contai... [v7] |
| 6 | LOC_Os12g09240 | 8.5 | 0.003551 | NBS-LRR disease resistance protein, p... [v7] |
| 7 | LOC_Os07g33690 | 8.5 | 0.01496 | NBS-LRR type disease resistance prote... [v7] |
| 8 | LOC_Os11g45970 | 8.5 | 0.01496 | NBS-LRR disease resistance protein, p... [v7] |
| 9 | LOC_Os02g55540 | 8.333333 | 0.003283 | F-box/LRR-repeat protein 14, putative... [v7] |
| 10 | LOC_Os11g43420 | 7.5 | 0.00617 | LZ-NBS-LRR class RGA, putative, expre... [v7] |
| 11 | LOC_Os12g28100 | 7.5 | 0.025781 | NBS-LRR disease resistance protein, p... [v7] |
| 12 | LOC_Os07g40810 | 7 | 0.008151 | NBS-LRR type disease resistance prote... [v7] |
| 13 | LOC_Os01g05600 | 6.5 | 0.010787 | NBS-LRR disease resistance protein, p... [v7] |
| 14 | LOC_Os11g13940 | 6 | 0.014306 | NBS-LRR disease resistance protein, p... [v7] |
| 15 | LOC_Os01g71106 | 4.5 | 0.033894 | NBS-LRR disease resistance protein, p... [v5] |
| 16 | LOC_Os04g30320 | 4.5 | 0.033894 | F-box/LRR-repeat protein 14, putative... [v7] |
| 17 | LOC_Os04g40800 | 4.5 | 0.033894 | OsFBL18 - F-box domain and LRR contai... [v7] |
| 18 | LOC_Os06g50280 | 4.5 | 0.033894 | OsFBL31 - F-box domain and LRR contai... [v7] |
| 19 | LOC_Os06g10300 | 3.73913 | 1.98E-05 | OsFBL29 - F-box domain and LRR contai... [v7] |
| 20 | LOC_Os08g35900 | 3.684211 | 0.000132 | OsFBL49 - F-box domain and LRR contai... [v7] |
| 21 | LOC_Os11g44960 | 3.444444 | 0.013905 | NBS-LRR disease resistance protein, p... [v7] |
| 22 | LOC_Os03g48890 | 3.285714 | 0.000346 | LRR receptor kinase, putative, expressed [v7] |
| **Resistance in BR2655** | | | | |
| 1 | LOC_Os01g72390 | 21 | 0.002569 | NBS type disease resistance protein, ... [v7] |
| 2 | LOC_Os08g14850 | 18.5 | 7.4E-05 | resistance protein, putative, expressed [v7] |
| 3 | LOC_Os01g42380 | 14 | 0.017622 | pleiotropic drug resistance protein, ... [v7] |
| 4 | LOC_Os11g45620 | 13 | 0.000311 | rust-resistance protein Lr21, putativ... [v7] |
| 5 | LOC_Os11g37850 | 12 | 0.002282 | stripe rust resistance protein Yr10, ... [v7] |
| 6 | LOC_Os03g11340 | 11.5 | 0.000696 | leucine-rich repeat resistance protei... [v7] |
| 7 | LOC_Os07g04900 | 10.5 | 0.001194 | NBS-LRR disease resistance protein, p... [v7] |
| 8 | LOC_Os07g19320 | 10.5 | 0.001194 | stripe rust resistance protein Yr10, ... [v7] |
| 9 | LOC_Os01g58520 | 10 | 0.000889 | disease resistance protein, putative,... [v7] |
| 10 | LOC_Os11g37880 | 9.5 | 0.002055 | stripe rust resistance protein Yr10, ... [v7] |
| 11 | LOC_Os12g32670 | 9.5 | 0.008712 | CC-NBS-LRR resistance protein, putati... [v7] |
| 12 | LOC_Os01g06870 | 9 | 0.0027 | resistance protein SlVe1 precursor, p... [v7] |
| 13 | LOC_Os04g53496 | 8.571429 | 4.68E-06 | NBS-LRR disease resistance protein, p... [v7] |
| 14 | LOC_Os12g09240 | 8.5 | 0.003551 | NBS-LRR disease resistance protein, p... [v7] |
| 15 | LOC_Os07g33690 | 8.5 | 0.01496 | NBS-LRR type disease resistance prote... [v7] |
| 16 | LOC_Os11g45970 | 8.5 | 0.01496 | NBS-LRR disease resistance protein, p... [v7] |
| 17 | LOC_Os12g28100 | 7.5 | 0.025781 | NBS-LRR disease resistance protein, p... [v7] |
| 18 | LOC_Os07g40810 | 7 | 0.008151 | NBS-LRR type disease resistance prote... [v7] |
| 19 | LOC_Os08g42670 | 7 | 0.008151 | resistance protein, putative, expressed [v7] |
| 20 | LOC_Os02g47230 | 7 | 0.009374 | Disease resistance/zinc finger/chromo... [v7] |
| 21 | LOC_Os01g05600 | 6.5 | 0.010787 | NBS-LRR disease resistance protein, p... [v7] |
| 22 | LOC_Os11g13940 | 6 | 0.014306 | NBS-LRR disease resistance protein, p... [v7] |
| 23 | LOC_Os12g13730 | 6 | 0.014306 | pleiotropic drug resistance protein 2... [v7] |
| 24 | LOC_Os01g42350 | 5.857143 | 0.00052 | pleiotropic drug resistance protein, ... [v7] |
| 25 | LOC_Os03g40194 | 5.5 | 0.019016 | disease resistance RPP13-like protein... [v7] |
| 26 | LOC_Os07g09900 | 5 | 0.025347 | disease resistance protein RPM1, puta... [v7] |
| 27 | LOC_Os11g12340 | 4.714286 | 0.00365 | disease resistance protein RPM1, puta... [v7] |
| 28 | LOC_Os01g04070 | 4.5 | 0.033894 | verticillium wilt disease resistance ... [v7] |
| 29 | LOC_Os10g22930 | 4.444444 | 0.001739 | Cf2/Cf5 disease resistance protein, p... [v7] |
| 30 | LOC_Os01g42410 | 4.25 | 0.004556 | pleiotropic drug resistance protein, ... [v7] |
| 31 | LOC_Os04g11780 | 3.8 | 0.043307 | resistance protein LR10, putative, ex... [v7] |
| 32 | LOC_Os09g14490 | 3.666667 | 0.008828 | TIR-NBS type disease resistance prote... [v7] |
| 33 | LOC_Os11g44960 | 3.444444 | 0.013905 | NBS-LRR disease resistance protein, p... [v7] |
| 34 | LOC_Os04g13210 | 3.2 | 0.016376 | multidrug resistance-associated prote... [v7] |
| 35 | LOC_Os12g11370 | 3.142857 | 0.048883 | verticillium wilt disease resistance ... [v7] |
| 36 | LOC_Os05g31550 | 3.111111 | 0.027194 | disease resistance protein RGA4, puta... [v7] |

**Supplementary Table 4:** Reference assembly parameters

High quality clean reads were mapped to the rice reference genome Rice Genome Annotation Project 7 (RGAP7) (<http://rice.plantbiology.msu.edu>) using reference assembly tool of CGWB. The mapping parameters are presented below:

| 1 | Reference type: | Genome annotated with genes only |
| --- | --- | --- |
| 2 | Reference sequence: | *Oryza sativa* (ssp japonica cv Nipponbare) sequence |
| 3 | Gene track: | *Oryza sativa* (ssp japonica cv Nipponbare)_Gene |
| 4 | Mapping type: | Also map to inter-genic regions |
| 5 | Mismatch cost: | 1 |
| 6 | Insertion cost: | 1 |
| 7 | Deletion cost: | 1 |
| 8 | Length fraction: | 0.5 |
| 9 | Similarity fraction: | 0.5 |
| 10 | Global alignment: | No |
| 11 | Auto-detect paired distances: | No |
| 12 | Strand specific: | Both |
| 13 | Maximum number of hits for a read: | 1 |
| 14 | Count paired reads as two: | No |
| 15 | Expression value: | TPM |
| 16 | Calculate RPKM for genes  without transcripts: | No |
| 17 | Create report: | Yes |
| 18 | Create fusion gene table: | No |
| 19 | Create list of unmapped  reads: | No |

**Supplementary Data 1:** Eight blast Resistance Protein sequences

**RP1_857**

>EMBOSS_001

MDSGRWISAMVSSVGWKAGDPVLRRYGHATIQAYRRTLHVRLLWLVDNGPWLHLSNLTTTDYGLASFWLYELKKALTDADALLENWRCEILRAESIGSRGTATKVRRTFSGVLDPLKVIPRHSKEFDVALQEELADVFVSIVGMGGIGKTTLAQMVYDDDIVQQHFPVRLWVTVSEDFDTTTILQGIVGPSIQGTSLLQLHVREVLCGKQFLLVLDGVSRFNAEKWDRLKISLECCGLGSAVLMTTRSENKSSTSRAYYLGKLADEYVWSIAKQIAFVKEEEGQDLVHIKEAVVTISDGIPLSAAILGGLLRSRLYCELDDWLVSWADACEERSVWRIELHGEWCQSDAQREDIVFLAIELSYKHLPACIKGCFAFCSLFPRTHKIDKDMLIQLWMANDLIPYDDAMDLEAKGSWIFDELVSRCFFQVTKRAQPSQSNRTKWRMHDLVHDTAVLISNVEFTTVLSSVMFGSPHVQSLHHMSIVSCRNKVTCIPLLPAPNLPNLRTLLSLEEQYPLYEWNVDFSKCKSLRVLDLHGFHSSQVMLPSRFLEHLRYLDLSNSWITSIPDDVVYLYNLQTLRLSECCYLKQLPKDLRKMKSLRNLYLDGCFRLENVPLNLGQLKDLHILTTFIVGTDDGCGIGQLKGLNLEGQLEIYNLKNVKRIEDVKGVNLHTKENLRHLTLCWGKFRDGSMLAENANEVLEALQPPKRLQSLKIWRYTGLVFPRWIAKTSSLQNLVKLFLVNCDQCQKLPAIWCLKTLELLCLDQMKCIEYICNYDTVDAEECYDISQAFPKLREMTLLNMQSLKGWQEVGRSEIITLPQLEEMTVINCPMFKMMPATPVLKHFMVEGEPKLCSSYVL

**RP2_1492**

>EMBOSS_001 MDTGIIVARWVVGKALNPVLDGLVEAWAASQKLGPNVDALKMELLYARAMLNNVRGREIHNTDLNELLQKLRDLAYNADDVLDELDYFRIQDELDDTSEAAAEHAKGCVSDLFLNAHHTAKAAGKLLGFSSSCSSCATNNGPGDSITAACCGSPHNTIHAIGKRLCFSTSLVDDCDHDYGCVHDERDHVKGKSTPKLKFDRVGLSKKMKIIVEQLQPVCAKVTAILNLELMGSHLSIESSTAKSRPITAPTSIEPTLYGRDAVMKRIIDSITQGTCCEEYLTVLPIIGPGGIGKTTLIQHIYNSQQVNHFQIMVWTCVSQSFSVDKLIEEIKEKLPSVEGEKKGSAEELIVQRLKSKRFLFILDDIWKCESDDWKRLLVPLRKGQTKGNIIIVTTRFLVVTETVKTSDNKIQLEGIDDEAFWELFLAYVFGPEKSKNDKDLLCIGKDIVKKLKGSPLAAKTVGKLLSNHLDRVHWMRVLDSKEWELQAGDHDIMPALKLSYDYLPFHLQQCFSYCALFPEDYKFNNKELIRFWIGLDILHSESQNKAFEDIALSNIDSLVSHGFFKREETDGHPCYIIHDLLHNLALKVASLECVSLHSSNVKSVEIRPSIRHLSIITDGANDTDGITDENFKSELIKLKKRLKVENVQTLMIFGEVDKSFIGCFHDLFKEASALRVLYFPKMPFAVGSILDEFTTLVHLRYLRLGTTIGNNFHLPINLSRFYHLRILDLEKWDNCFHLPGDISNLAKLHNFLVPGYPIHSNISNVGKLQFLQELKGFQVNRKDVGFELKQLGYLMELRELRIDNLEKVHTKEEAAEAKLLSKTRLRKLELNWKQGRTSTNAFNEDQILEKLQPHSSLQELSIHGHGGSSCPKWLGTELSVKFLETFRLKNVVWNILPPLGEVFLVGGPGEESIGQKTSQNFRSLKRLELVKLPNLRKWVAKEIFPTFFSVLEVLIVRKCNELAELPFSYHTYCTSEEDVKATCFPRLRELEIHNCPKIVSLPPIPYTQTLCSVNITDVGTGLESLVYSSKSSKLEIKGNKDLKVLDDNVFASRNLHKLQNLTIEGFPPLEERHLQMLTSLKRFSLFSSSIAFNPTVERSDVEWRLSIENLMIQDWNGSGKELTQLLFHLPKLSLLSLGGCRKKTLLSIALTQQQTSAQVESTQVTASNHRQQQKAEDLDLLEEEEVTQLDVDGEDEDDDRLLLTNSLEQLWIVNYKELILVSHPLPIGHHNKEEEGTGGGWGLQALCSLRQLGIRGCPLLLSAYEAPACLFPSSLQYLQITGPMEGVQMLDLSNLTSLTKLFIEDCGEYLRKGLLPLLAQGQLSNLIVYKTYGLFAGVLDSILRGAQEEQEQLHLLEHSSKLRVLETDDLAGILVKPICRLLSSSLTNLTLQGNSEVKRFTNEQEEALQLLTFLQDLKFIHYDKLRCLPAGLHRLTNLKRLMIMNCPSIQSLPKDGLPGSLKYFIVRDNEKLVKQCKKLKKTNPEIELIL

**RP3_1480**

>EMBOSS_001

MERQTGSVLGTSLRREYPGLVKEYAGPGQRKRKRLAMSWGHYFGEKDHNGMTAGNRVKDEFWSFFTTQEEDMEELDKNIDNYCQARVPKIICQARVDAMKKYYGKGIKGKNASAIELNFEQYMTCKLDWINVDAWKCFCHWWTSDKYKEKRKRGQDARFANEDYAQQQGGSLPFCTIQQNLVYQLGPENASGLDTYRVQMAGFKASLKGSGQIRSEKVKQRINIYCQVYHEERGEEHQPVSSELDGNVVYKAFGGLKHGRFAMGNGVFKKTEVLAAVKHKKSGISGSTNSYNAVVRENAQLRHEVTEQRGMIREQRGMIQEHRGMLKKNPISKFNLHAYRQYMRSLEWTYLRRYFQGGSHKERLSIDVLIIHPFVDLFCGGNPVTKVDGPPPSGRHNLATLVGGAGAGAASAGKECNSNAASRGQYQNFHEIPPDDADFNNLTIYLKSASPAQINFKFWFFCSLQFMMASSLFSYFFGMKSRILSPALPQQSYLSSAELPSLTDHVNEEVAKLDRTVRRITAVLVDADEREIADETMKLWISELKQVTWEAEGILEDYSYELLRSTTVQEEKNILDRISKVRKFLDEICRDRVDLGLIDQEGLCRKESRISRCTSSLLDPLEVYGREDEKKLIISSLLDGCLTFKKRRLKEHEYETCKAGAVRLISIVAMGGMGKTTLARLVYNDARVQNHFDIQAWVWVSEVFDEVRLTKAAIESVTAKPCDLTELEPLQRQLHEEVKGKKILLVFDDVWNEDTIKWETMKRPFSAVATGSHMIITTRNENVSTIVQAKKVIHLGGLQKDDSWALFCKLSFPDNACRETELGPIGRKIVEKSDGVPLVLKTLGAMLSLDTSLEFWNYVLTSDLWELGPGWDHILPILKLSYYSLPAILKRCFTFLAAFPRGHKFDLEELVHMWCALGFIQEDGVKRMEEIGHLYVNELVRRSFLQNLQLAGSREKFVIVHDLIHDLAKSIGGKEILVKKCCGSSVGGCNTSANNHLRYLAVLVGTTPFYSDNKLVPFTLPVAGHFPLRSLSFQSKWRTYLRSCVRNNLRTFFQVLVQSQWWYNLEGCLLHSPHLKYLRILDVSSSDQIKLGKSVGVLHHLRYLGICQREIPEAICKMYKLQTLRNTYPFDTISLPRNVSALSNLRHLVLPREFPVTIPSGIHRLTKLQSLSTFAVANSGSGAATLDEIKDINTLQGQLCIMDLQNITHDRIWEPRSANLSKKKLTRLELVWNPLPSYKSVPHDEVVLESLQPHNYIRQLVISGFRGLNFCSWLGDRSLFSLQELELCKCYYTDHLPPLGQLPNLKQLKLTSLWKLRSIGPEFYGDCEAPFQCLETLVVQNLVAWEEWWLPENHPHCVFPLLRTIDIRGSHKLVRLPLSNLHALAGITVSSCSKLETIVGLKERCEVTAGNGGLQAGQTNVLPSLRRVKITACPSLEEPLISMLRRQTEIGFSYWEQSPSTSPTDIADFGKTPWKDSGKV

**RP4_1080**

>EMBOSS_001

MAAAAAMSTIPRTPAVTAASLPPAAGSRSSCGGVVGVRVPNLHRQRFPRPRSSAPWASSRSSIRILIIILRARSVSPRSGLNRFVESFDKFDVQGIVMEEAILILGVDEELKELQRRMKQIQCFLHDAEQRRIEEEAVNNWLGELKNAIYDADDIIDMAKFEGSKLLANHSSLSPLPIKYISCCNLSVTSCVRNVWTHRKIALQIRRVNYNLQRISIDKTFLALENVKATYRVLAPSKRHTSHLVEPNLVGKEIKYATSRLVEMILTHREEKAFKVAIVGTGGVGKTTLAQNIYNDQRVKGNFSKHAWICVSQEYSEVNLLKELLRNMGVHERQGETVGELQSKLASTIKDESLFVVLDDVWQSEVWTNVVRTPFHDAAKATILVTARDELVVRRVGAEHLHRVEMMSTDVGWELLWKSMNIKEEKEVETLQHIGTKIVSKCGGLPLAIKVIASVLATKEKTKNTWEKVIESSAWSMSKLPAELRGALYLSYDDLPHNLKQCFLYCALYVEGQMMHHADLVRFWVAEGFVEEQEGQLLEDTAEEYYHELICRHLLEPDPFYFDHYRCKMHDLLRYLAQHLSREECYFDQLPLEPTTWSKLRRISIVNKTDMLSSVVEKGHCRVRTLMFCMSPNIDSDVFMRFPHLRVLDLTGSIVQRIPDSINSLIHLRLLDLDATDISCLPDSIGSLTNLQILNLQRCYALHDLPMAITKLCSLRCLGLDDTPINQVPRGINKLSLLNDLQGFPVGHSYVNTRKQDGWNLEELGHLSEMKRLGMIRLENAMPCGTSSLLDKKHLKFLNLRCTTHTKESYTMEDITNIENVFDELKPPCNLEDLSIAGSFGQRYPTWLGADLSSLKILRLIDCASWAHLPAVGQLPNLKCLKIMGASAVTKIGPEFLCDKTATPRFLGTIAFPKLEWLVISDMPNWEEWSFTEEVVGASDGKSCTENNKMVLQVMPLLQKLELGDCPKLRALPQQLAQATSLKWLHIERAQALKVVEDLTFLSDSLLLSKCEGLERLSNLPQVRTLYVSECPALRWAQKLDCVQQLWLSKDLQMEFPLWLSLLKQRYQQLHGEELDLYTW

**RP5_1274**

>EMBOSS_001

MTELASGAVSSLLVVIRNEAALLGGVRDDVRLLAQDCNNCIDLYLYSGNPEIHRAKGRLRRHLWWAYWYLRKMVAQHRAAIQLCQLKDRARDVGERRMRYGVEVPATTKAAAPDAEGGYAAGDDEDYEDHSARRAVYEPPALEDYVNAKLLEWAEEIPPDAIETLSIAIVAPDTDNKEVLDLARDTLVVPGNYYHRSITVNVPAVHADFLPLRPKEILYYILRELEPGSQKHVTYQGDPWQDYYNIYCGKKRVLHKIKRNIEKMNVYKKLEKIKSDIKDGQHKSDKQLLLQLQKKGVDQVDLHVLVQLLLLQSQQDQANNKAVDMYKLPEWNYDNIMKIARKLKKHMEADEELNEQISVEKRTTKQGGGGENKEEKDKGDGDREEGKEENEKDKGEADGEGEQEEEMKDKEKGGEERKEEPHHEKQEHKKEKEVKEEERKEEQNKEKEEREGTEEKQVGGGEKKEEKEDGDDDGVIDDNYDEEEEEEEEDDDPIHLHEAQYVQILLEVFPKITSSKAQQQDKSEAKQATNTATTTLDEVRIKQMINDAKRDILRELRGGKSDKNEATGEPDVPPDKNQPTGEHAGVPDQNGEACFEELELKIEEIKQELKEQLKIKGIVDKIKHHLQGECPLIILKVDDMMDGSRWEEFRRALSLLECSADALIFTTESTEQAKGYCYPPREPIDHCSHVGLYHHTVLELTSKQKNKSNYDPRIFLDILKECEGHEFCMRIFTHAVYANPKKSTEELSKLHSTLQDSQKSFDAIAKNMLMYSYNDLPKEYKSCLLYLAIFPKGQKIRRSTLIGRWVTEGLTFKEDWPSSVRQANQCFDALIRRWLVYPADIGATGKIKSCEVGDLVHGFITTIARKQHIVETRLSHHLARHFSIFNDLRLRSSDRIQTFFQSLSQSSRVSLLKVLDLEGCQCFGVKNQRYLKDICNKMLLLKYLSLKGTNITQLPREINCLRELEVLDIRETMVPANATVNVLLLKLKRLLAGHIDPSLRNFRTSVHIPHRIDKMVNIEVLSNVKAQRSDDLEDIGKLWQLRKLGVVINAKKSHLEKLLKAISDLHECIRSLTITIPTTTLEGTPSNPELPDHIDSNLPHPKKLESLSISGARYLFPLLIKSDNNKLAKVTLSSTQLNQDDLEVLAKLPKLQCVRLRHISCTESMLIFKKDDFKCLKYLLIEGFNLTNITFENRSACELEKMVLSSTSIESISGVDRLPKFKELELNNSPVPNEYTVFLFLKKQILCSMLDWILKLNDELGKWGKQSFMQIKYA

**RP6_1033**

>EMBOSS_001

MATIVDTLVGSCINKLQAIITDKAILILGVKDELEELQRRTDLIRYSLQDAEARRMKDSAVQKWLDQLRDVMYDVDDIIDLARFKGSVLLPNYPMSSSRKSTACSGLSLSSCFSNIRIRHEVAVKIRSLNKKIDNISKDDVFLKLSLTQHNGSGSAWTPIESSSLVEPNLVGKEVVHACREVVDLVLAHKAKNVYKLAIVGTGGVGKTTLAQKIFNDKKLEGRFDHRAWVCVSKEYSMVSLLAQVLSNMKIHYEKNESVGNLQSKLKAGIADKSFFLVLDDVWHYKAWEDLLRTPLNAAATGIILVTTRDETIARVIGVDRTHRVDLMSADIGWELLWRSMNIKEEKQVKNLRDTGIEIVRKCGGLPLAIRAIAKVLASLQDQTENEWRQILGKNAWSMSKLPDELNGALYLSYEVLPHQLKQCFLYCALFPEDATIFCGDLTRMWVAEGFIDEQEGQLLEDTAERYYHELIHRNLLQPDGLYFDHSRCKMHDLLRQLASYLSREECFVGDPESLGTNTMCKVRRISVVTEKDIVVLPSMDKDQYKVRCFTNFSGKSARIDNSLFKRLVCLRILDLSDSLVHDIPGAIGNLIYLRLLDLDRTNICSLPEAIGSLQSLQILNLQGCESLRRLPLATTQLCNLRRLGLAGTPINQVPKGIGRLKFLNDLEGFPIGGGNDNTKIQDGWNLEELGHLSQLRCLDMIKLERATPCSSTDPFLLSEKKHLKVLNLHCTEQTDEAYSEEGISNVEKIFEKLEPPHNLEDLVIGDFFGRRFPTWLGSTHLSSVKYVLLIDCKSCVHLPPIGQLPNLKYLKINGASAITKIGPEFVGCWEGNLRSTEAVAFPKLEWLVIKDMPKWEEWSFVEEEEVQEEAAAAAKEGGEDGIAASKQKGEEAPSPTPRSSWLLPCLTKLDLVGCPKLRALPPQLGQQATNLKKLFIRDTRYLKTVEDLPFLSGGLQVEGCEGLERVSNLPQVRELFVNECPNLRHVEELGGLEQLWLDEGMQEISSLWVPRLQEQHRQLHGDEHELEVTEWL

**RP7_1031**

>EMBOSS_001

MATIVDTLVGSCINKLQAIITDKTILILGVKDELEELQRRTNVIRSSLQDAEARRMEDLVVEKWLDQLRDVMYDVDDIIDLARFKGSVLLPDYPMSSSRKSTACSGLSLSSCFSNIRIRHEVAVKIRSLNKKIDNISKDEVFLKLNRRHHNESGSAWTPIESSSLVEPNLVGKEVIRACREVVDLVLARKKKNVYKLAIVGTGGVGKTTLAQKIFNDKKLEGRFDHHAWACVSKEYSRDSLLRQVLRNMGIRYEQDESVPELQRKIKSHIANKSFFLVLDDVWNSEAWTDLLSTPLHAAATGVILITTRDDTIARVIGVEHTHRVDLMSADVGWELLWRSMNINQEKQVQNLKDIGIEIVRKCGGLPLAIRVIATVLASQEQTENEWRRILGKNAWSMSKLPRELSGALYLSYEVLPHQLKQCFLYCALFPEDETILRDILTRMWVAEGFIDEEKGQLLEDTAERYYYELIHRNLLQPDGLYFDHWSCKMHDLLRQLACYLSREECFVGDVESLGTNTMCKVRRISVVTEKDMMVLPSINKDQYKVRTYRTSYQKALQVDSSLFEKLTYLRVLDLTNSHVQRIPNYIENMIHLRLLDLDGTDISHLPESIGSLQNLQILNLQRCKSLHRLPLATTQLCNLRRLGLAGTPINQVPKGIGRLKFLNDLEGFPIGGGNDNTKIQDGWNLEELAYLPQLRQLGMIKLERGTPRSSTDPFLLTEKKHLKVLNLDCTEQTDEAYSEENARNIEKIFEKLTPPHNLEDLFVGNFFGCRFPTWLGCTHLSSVKSVILVDCKSCVHLPPIGQLPNLKYLRINGASAITKIGPEFVGCWEGNLRSTEAVAFPKLEMLIFKEMPNWEEWSFVEEEEVQEEEAAAAAKEGGEDGIAASKQKGEEAPSPTPRSSWLLPCLKQLQLVECPKLRALPPQLGQQATNLKKLFIRDTRYLKTVEDLPFLSGCLLVERCEGLERISNLPQVRELRAGGCPNLRHVEELGGLEQLWLSKNMQKISSLWVPGLEEQHRQLHGDEHKLEVNE

**RP8_902**

>EMBOSS_001

MASSSSGVMNSLLTKLATLIREECYSKLKGVRNEVVSLEGELRSMEALLEKLACMDELDVQVKEWRDQVREMSYDIEDCIDDFVHRLGKYDVRSGLIKKTTELPRKLRARHQIAKKIEEIKNHVKEVNERRMRYKLDEYTSKSSCEPIDPRVVTIYANTADLVGIDIPRDDVVKLLMGDDEQQLKGFGGLGKTTLANEVYRKLDGHFHCRAFVSVSQRSDITRLMSKVLSELTGQYNLHIGELDNLLKVIREYLQDKRYLFMCRYFIVIDDLWDSSAWNVIRCAFPENNHGSRVLTTTRIYSVAISCCSNKKEYVYNMSPLGEVDSRRLLFSRIFGTGEACSEVFEEISGDILKRCGGLPLAIMSISSLLAGQSKTKWEYVRNSLGSMFERNPTLEDMKHILDFSYRNLPQHLKTCLLYLSIYPEDHTIERNDLVRQWMAEGFVSRTHGLDSEDVAQSYFNELINRSMIQPVQVDYNDEVLSCRVHDIMLDFIRSKSAEENFIVVLDHPQVVVGAHKKIHRVSVQYDADEEHGIISTTILGSLSQVRSIAVFRSSFRPSLLELKHLRVLHLELPMREVMDLTGICGLSLLRYLKIRGYYACFKLGMKIRQLLHLETFDLGESFVPRIAIPSDIVHLPCLLHLVIPCGTTLPDGIGSLKSLRTLTSLDLALNSVNNIECLGELTNMRHLGVHLGDIGLLADADVARRLDALCSSLESLSRSSSCLRSLDLDSHFILVSFDRLSDLCPSPRHLRRLNLYGCRLPRIPRWISQLHNLYSLLPSLVQLDLGIRECPKERIVVSGAGTAFRALRDLTFSCPKPRLAFLVGAMPRLQRLDLRFYVNGWEQQGGTCLPVGIENLPSSGLKIHLVNVFTDSGSQDVISAKSVLRRTFEVHQPSADLIFHF

**Supplementary Data 2:** RNAseq Experiment Overview


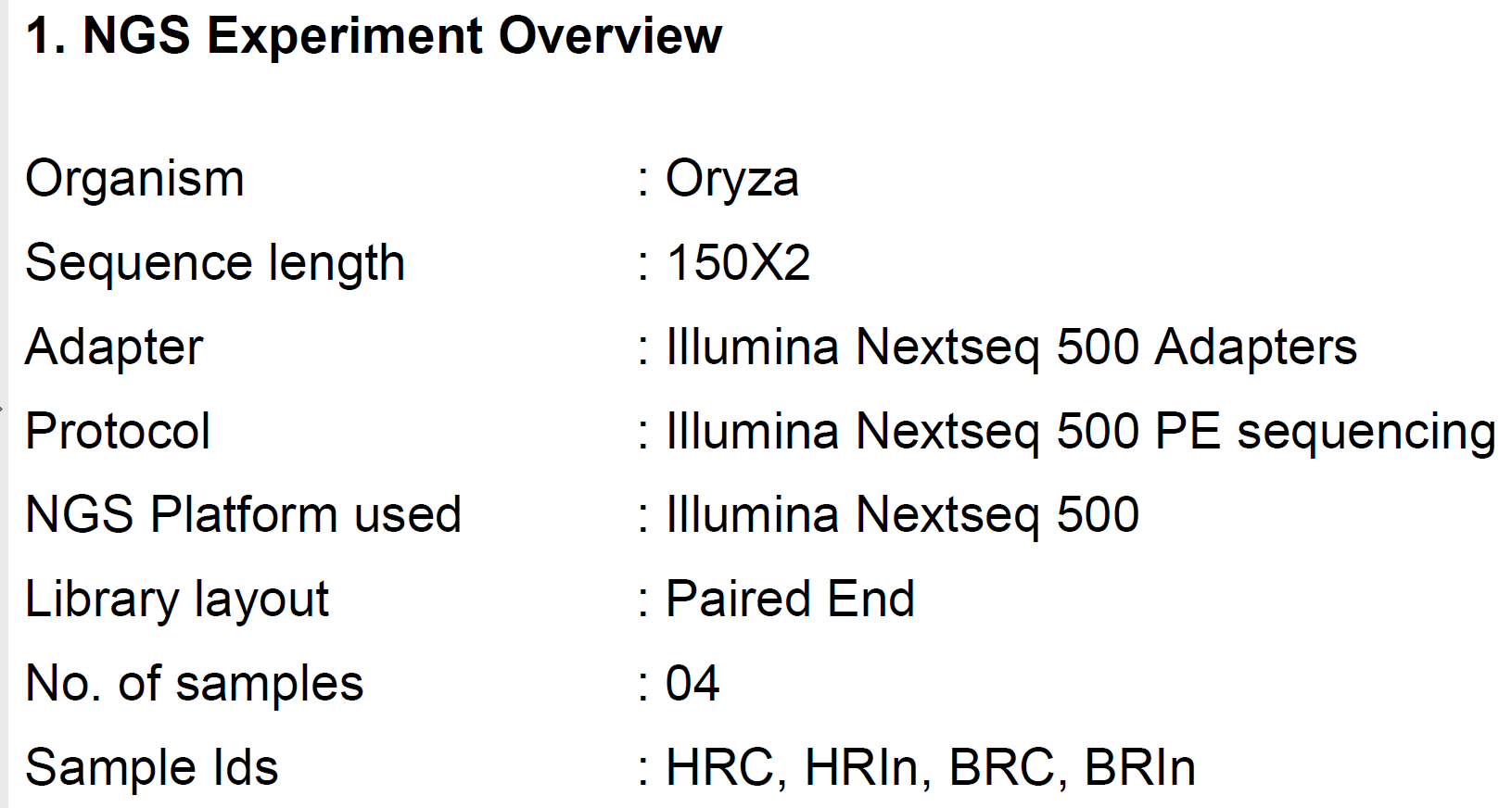


**Supplementary Data 3:** Summary of four sample data sets from RNA libraries obtained for rice varieties BR2655 and HR12 (C: Control and IN: Inoculated)


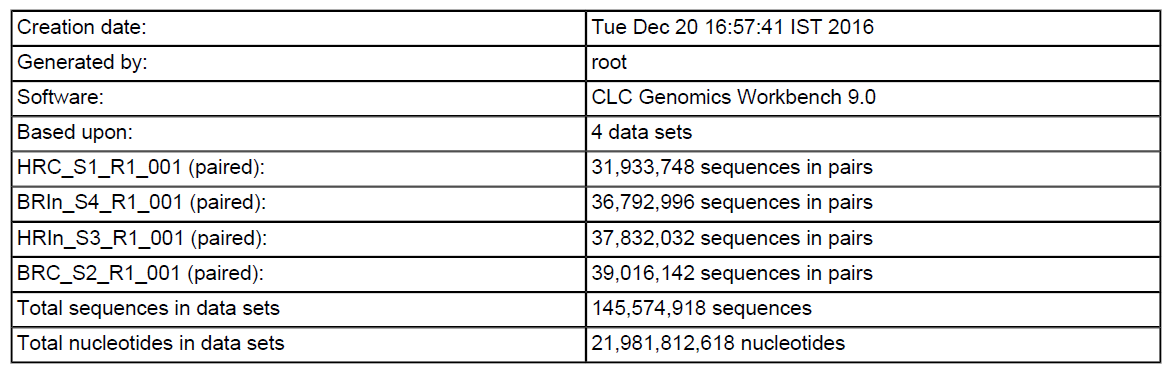


**Supplementary Data 4: Reference assembly**

Reference-based transcriptome assemblies of BR2655 and HR12 RNA-seq data were performed employing tool pipeline using the rice genome sequence as reference. The assembly of mapped reads resulted in the identification of a total of 34046 transcripts in BR2655 Control and 32682 transcripts in BR2655 inoculated. Similarly, 32173 transcripts in HR Control and 36058 transcripts in HR inoculated cultivars.
